# Involvement of HHV-4 (Epstein–Barr Virus) and HHV-5 (Cytomegalovirus) in Inflammatory Bowel Disease and Colorectal Cancer: A Meta-Analysis

**DOI:** 10.3390/cancers14205085

**Published:** 2022-10-17

**Authors:** Luigi Marongiu, Sascha Venturelli, Heike Allgayer

**Affiliations:** 1 Department of Experimental Surgery—Cancer Metastasis, Medical Faculty Mannheim, Ruprecht-Karls University of Heidelberg, Ludolf-Krehl-Str. 13-17, 68167 Mannheim, Germany; 2 Department of Internal Medicine VIII, University Hospital Tübingen, Otfried-Mueller-Strasse 10, 72076 Tübingen, Germany; 3 Department of Biochemistry of Nutrition, University of Hohenheim, Garbenstraße 30, 70599 Stuttgart, Germany; 4 Institute of Physiology, Department of Vegetative and Clinical Physiology, University Hospital Tübingen, Wilhelmstr. 56, 72074 Tübingen, Germany

**Keywords:** colorectal cancer, inflammatory bowel disease, Epstein–Barr virus, cytomegalovirus, meta-analysis, viral infection

## Abstract

**Simple Summary:**

The prevalence of colorectal cancer (CRC) and inflammatory bowel disease (IBD) is increasing worldwide. Understanding what factors foster these diseases’ development can help reduce their burden. Viral infections have been suggested to be involved in the genesis of these diseases, but the data is contradictory. The present study analyzed 11,413 articles on the topic and identified 196 that could provide information on the relationship between a viral infection and these gastrointestinal diseases. The Epstein–Barr virus (EBV, HHV-4) was strongly associated with IBD, and cytomegalovirus (HHV-5) with both CRC and IBD.

**Abstract:**

Gastrointestinal diseases (GDs) include colorectal cancer (CRC), gastric cancer (GC), and inflammatory bowel disease (IBD). CRC and GC are typically diagnosed at later stages of development, reducing patients’ chances of survival. IBD is characterized by chronic intestinal inflammation and is a significant risk factor for the development of CRC. Chronic bacterial infections have been shown to promote some GDs, but the role of viruses in the etiology of these diseases is less clear. The present meta-analysis retrieved literature on the viral prevalence in GD patients, measuring the GD risk in odd ratios. By quantifying the study heterogeneity, the literature bias was fundamentally included in the analysis. The analysis also included 11 metagenomic studies. Our meta-analysis retrieved 11,413 studies, with 196 suitable for analysis. HHV-4 (Epstein–Barr virus) was identified as a significant risk factor for the development of IBD, and HHV-5 (cytomegalovirus) as a risk factor for both CRC and IBD. Polyomaviruses and the Hepatitis B virus were also, less strongly, involved in the risk of CRC and IBD. No relations withstanding the literature bias were identified for GC. The study discusses these findings, as well as the role of other viruses in the etiology of CRC and IBD.

## 1. Introduction

About 15% of all human cancers have been shown to have a microbial etiology [1]. Of the eleven micro-organisms recognized as class one carcinogens by the International Agency for Research on Cancer (IARC), seven are viruses: Human Herpesvirus 4 (HHV-4, also known as the Epstein–Barr virus), the Hepatitis B virus (HBV), the Hepatitis C virus (HCV), Human Herpesvirus 8 (HHV-8, also known as the Human Kaposi sarcoma virus), Human Immunodeficiency virus genotype 1 (HIV-1), the high risk genotypes of Human Papillomavirus (HPV), and the Human T-cell lymphotropic virus genotype 1 (HTLV-1) [2]. In some cases, the link between a certain virus and a given type of cancer is strong, in terms of both epidemiological data and experimental modeling. For instance, HPV have been recovered in virtually all cases of cervical cancer and the oncogenic process is well characterized at the molecular level [3]. In other cases, such relationships are less evident: HPV has been recovered only in 13–56 percent of oro-pharynx cancers and in 0–84 percent of tissues derived from the cancer of the colon and rectum, indicating that other causes are involved in the development of these typologies of cancer [4,5]. The reverse is also true: some types of cancer are not easily associated with microorganisms with transforming potential, even if the data are tantalizingly suggesting a relation. Among these, are the malignant diseases affecting the gastrointestinal tract.

Cancer of the stomach (better known as gastric cancer, GC) and colorectal cancer (CRC) share some similarities, including a high number of deaths caused worldwide, detection (at least in the western world) at rather late stages of the tumor development, and risk factors such as hereditary mutations, obesity, and sedentariness [6]. CRC is the second most common type of cancer worldwide, with a death toll of over half million people, yearly [6]. CRC is a group of heterogeneous malignancies, rather than a single type of tumor: about two thirds of cases are known as sporadic, further subdivided into hypermutable (16%) and non-hypermutable cancers (84%). The remaining one-third is classified as hereditary (or familial) [7,8]. The prevalence of GC has been decreasing in recent years, but it still remains the third cause of cancer-associated death after lung cancer and CRC, with over a million new cases yearly [9].

Every person has a four percent life-time risk of developing sporadic CRC [7], but some factors can increase this basal value: for instance, diet, sedentary lifestyle, and alcohol consumption [10], which are also risk factors for the development of GC [11,12]. Chronic inflammation of the gut is also a risk factor for the development of CRC that goes under the general term of inflammatory bowel diseases (IBD) and can be further differentiated into Crohn’s Disease (CD) and ulcerative colitis (UC) [13,14,15]. Patients suffering from IBD, had a CRC risk increased by 40% in comparison to healthy people [16,17]. IBD incidences have escalated in the last few years and cause an economic burden of over six billion, in the US alone [18,19].

Recently, specific infections have been suggested to affect the risk of sporadic CRC [20]. For instance, the prevalence of a CMV infection has increased 350% in IBD cases, between 1998 and 2014, with indications of a higher mortality in the infected patients [21]. In the case of GC, an infection with *Helicobacter pylori* is known as a major risk factor, albeit the precise oncogenic mechanism is still poorly understood, and only a subset of *H. pylori* carriers develop GC [22]. It is believed that the abnormal activation of the NF-κB signaling pathway induced by this bacterium might lead to chronic inflammation and, eventually, to cancer [23]. An infection with *Fusobacterium nucleatum* might put further strains on the aberrant activation of NF-κB, further enhancing the gastrointestinal carcinoma risk [24] and a HHV-4 infection is observed in about 9% of cases [25]. Still, the situation is complex and hardly fits into a one cause-one effect model. In addition, most of the bacteria or viruses that have been associated with a higher risk of CRC, are commonly present in the healthy gut [26].

The idea of a relation between infection and CRC has been discussed in the last few decades [27]. Several experimental and epidemiological studies have provided support for the association between the presence of certain bacteria and CRC [28]. The current hypothesis is that several species of bacteria might be involved in CRC carcinogenesis and/or progression [29]. Moreover, the link between a viral infection and CRC remains less clear [26,30]. The odds ratios (OR) between infection with a given virus and development of CRC, ranged between 0.7 and 58.8 for HPV, 0.9 and 9.0 for the John Cunningham virus (JCV), 0.1 and 10.4 for Herpesviruses in different studies [31,32,33,34]. Furthermore, zur Hausen and collaborators have suggested the presence of a novel class of zoonotic vectors that might be involved in the development of CRC [35].

In relation with the establishment of the current paradigm linking oncogenic bacteria and CRC [29], bacteriophages (or simply phages) have been gaining interest in the most recent studies [36]. Using metagenomic approaches, research groups have shown differences in the prevalence of phages between the healthy and inflamed gut [37]. Specifically, an increased richness (number of species) of the phages belonging to the order *Caudovirales*, has been consistently reported [38].

Both CRC and GC develop rather slowly and symptoms are noticed only when the tumors have grown to a considerable size [10]. Thus, an early detection of the subjects at increased risk of developing either CRC and GC is paramount. To achieve such an early warning, a good comprehension of the risk factors that can facilitate CRC and GC carcinogenesis, progression or metastasis is fundamental. The present work had the objective to provide updated insights into the relationship between a viral infection and gastrointestinal diseases (CRC, GC, or IBD), in order to improve our understanding of their risk factors and to support the preventive measures.

Given the inconclusive data present in the public domain, we sought to provide an updated meta-analysis regarding the viral associations with CRC, GC, and IBD, with particular attention put on the phages. The research question posed herein was the following: *Is the presence of viruses associated with an increased risk of developing CRC/GC/IBD?* We answered this question by surveying the literature in the public domain and stratifying the eligible articles into groups that were used to determine the odds ratios (ORs) able to indicate the strength of such an association.

## 2. Materials and Methods

### 2.1. Protocol

The present study was conducted following the Preferred Reporting Items for Systematic Reviews and Meta-Analyses (PRISMA) [39]. The complete search query is provided in Supplementary File 1. The search was aimed at identifying an increased risk of CRC, GC, or IBD. CRC included both carcinomas and adenocarcinomas. The viral families and prototypical viruses included in the analysis were:

- *Adenoviridae*: Adenovirus (ADV).
- *Anelloviridae*: Transfusion Transmitted Virus (or Torque Teno virus, TTV).
- *Astroviridae*: Astrovirus (AstV).
- *Bunyaviridae*: members of the Orthobunyavirus genus (BV).
- *Caliciviridae*: Norovirus (NV), Sapovirus (SaV).
- *Herpesviridae*: Human Herpesvirus 1 (HHV-1 or Herpes Simplex virus), Human Herpesvirus 3 (HHV-3, or Varicella Zoster virus), Human Herpesvirus 4 (HHV-4, or Epstein–Barr virus), Human Herpesvirus 5 (HHV-5, or Cytomegalovirus), Human Herpesvirus 6 (HHV-6), Human Herpesvirus 8 (HHV-8, or Kaposi’s sarcoma-associated herpesvirus), Inoue–Melnick virus (IMV).
- *Hepadnaviridae*: Human Hepatitis B virus (HBV).
- *Flaviviridae*: Human Hepatitis C virus (HCV).
- *Matonaviridae*: Rubella virus (RuV).
- *Retroviridae*: Human Immunodeficiency virus (HIV), Human T-cell lymphotropic virus type 1 (HTLV-I).
- *Papillomaviridae*: Human Papilloma virus (HPV).
- *Parvoviridae*: Parvovirus B19 (B19), Bocaparvovirus (HuBV).
- *Polyomaviridae*: BK virus (BKV), John Cunningham virus (JCV), Merkel cell polyomavirus (MCPV), Polyovirus 5 (PyV6), Simian virus 40 (SV40).
- *Paramyxoviridae*: Measles virus (MeV), Mumps virus (MuV).
- *Reoviridae*: Reovirus (RV), Rotavirus (RoV).
- *Syphoviridae*: phages of the order *Caudovirales*.
- *Myoviridae*: phages of the order *Caudovirales*.
- *Inoviridae*: member of the Inovirus genus.

The viral families were clustered into the following groups:

- Herpesviruses (*Herpesviridae*).
- Respiratory viruses (*Adenoviridae*, *Bocaviridae*).
- Papillomaviruses (*Papillomaviridae*).
- Epithelial viruses (*Anelloviridae*, *Bunyaviridae*, *Matonaviridae*, *Paramyxoviridae*, *Parvoviridae*).
- Intestinal viruses (*Astroviridae*, *Caliciviridae*, *Noroviridae*, *Reoviridae*).
- Polyomaviruses (*Polyomaviridae*).
- Hepadnaviruses (*Hepadnaviridae*).
- Flaviruses (*Flaviviridae*).
- Retroviruses (*Retroviridae*).
- Phages (*Syphoviridae*, *Myoviridae*, *Inoviridae*).

### 2.2. Eligibility Criteria

The primary, original publications in international peer-reviewed journals, of human subjects, in the English language, and reporting the prevalence of viral infections in human patients were included.

### 2.3. Information Sources

PubMed, Cochrane Library, Web of Science Core Collection, Cinahl, and ClinicalTrial.gov.

### 2.4. Search

The search terms were subdivided into two groups: pathology and pathogen. These groups were combined with a logical AND operator. The terms were composed by the combination of the following terms.

- Pathology: “intestinal neoplasm”, “colorectal cancer”, “rectal neoplasia”, “Crohn’s disease”, “inflammatory bowel disease”, “ulcerative colitis”, “regional enteritis”, “granulomatous colitis”, “terminal ileitis”.
- Pathogen: “virus”, “giant virus”, “bacteriophage”, “Siphoviridae”, “Podoviridae”, “T7-like virus”, “φ-like virus”, “Herellevirus”, “Myoviridae”, “Tristromavirus”, “Bicaudavirus”, “Pycodnaviridae”, “Caudovirales”, “Ackermannvirus”, “Ampullavirus”, “Clavavirus”, “Corticoviridae”, “Cystoviridae”, “Fuselloviridae”, “Globulovirus”, “Guttaviridae”, “Inoviridae”, “Leviviridae”, “Lipothrixviridae”, “Microviridae”, “Plasmavirus”, “Pleolipovirus”, “Rudiviridae”, “Sphaerolipovirus”, “Tectivirus”, “Turrivirus”, “Polyomaviridae”, “Poxviridae”, “Simian virus 40”, “Papillomaviridae”, “Herpesviridae”.

### 2.5. Study Selection

In the first round of the assessment, the duplicated entries were removed; reviews, case reports, abstracts, clinical studies related to drugs or vaccines, and articles not written in the English language were also dismissed. In the second round, titles and abstracts were read; articles not related to a viral infection in the CRC or inflammatory diseases were withdrawn. In the third round, the whole articles were read and only those reporting the presence of viruses in intestinal lesions or metagenomic studies were included in the present review.

### 2.6. Data Collection Process

The publication repositories were queried with the strings reported in Supplementary File 1. The full texts of the selected articles were retrieved through the Central Library of the University of Heidelberg, Medical Faculty in Mannheim. Two investigators (L.M. and H.A.) selected the trials and independently reviewed the study titles and abstracts. The studies that met the inclusion criteria were retrieved for the full-text evaluation.

### 2.7. Data Items

The data related to articles, year of publication, number of patients having a pathology (cases) without infection, number of cases with infection, number of healthy patients (controls) without infection, number of controls with infection, tissue type, analytical method and virus species, were tabulated for further analysis. The articles were also subdivided into virome and non-virome studies. The studies were further stratified, based on the source of the data: the tissues were obtained from the colon-rectum, stomach (gastrointestinal tract), stools, sera, and medical (archival) records.

### 2.8. Risk of Bias in the Individual Studies

The assessment of the literature bias was measured, taking the study heterogeneity (variation of the degree of association between the cause and effect) into account, and quantified with the Higgins *I²* index [40]. The values of the *I²* index were stratified, as suggested [41]: low (<30%), moderate (30–60), substantial (61–75), and considerable (>75). The heterogeneity of the studies was quantified using the Egger’s regression test (ERT) of the funnel plot [42]. The ERT was preferred over the Cochran’s Q test (CQT) because it was reported to be more reliable on small data sets [43]. However, the ERT requires at least six publications and could not be computed in many instances. Thus, the CQT was included to supply the additional information on the study heterogeneity. The significant ERT and CQT indicate a publication bias.

### 2.9. Summary Measures

The odds ratio (OR) statistics were calculated for the non-virome studies, using the random effects model implemented by Mantel and Haenszel [44]. The trim-and-fill method was employed to adjust for the funnel plot asymmetry [45]; this method removed the studies causing the funnel plot asymmetry and replaced the omitted studies with the data around the newly computed center of the distribution. The trim-and-fill method required at least three data points for its computation.

### 2.10. Synthesis of the Results

The data were summarized in the tables reporting the OR and *I²* with their 95% CI and associated *p*-value. The classes that did not contain data were not reported in the table to increase the readability. The most relevant data were also displayed in forest plots and funnel plots to increase the understanding of the underlying relations between the studies [40,46].

### 2.11. Risk of Bias across the Studies

The risk of bias for the observational studies was assessed with the Newcastle–Ottawa scale (NOS) [47] by preparing a table with the following fields:

- Representativeness: if the selected article included more than 50 participants, a cut-off accepted to provide the statistically solid results, a positive value was assigned.
- Control group: if the selected article included a control group, a positive value was assigned.
- Documentation: if the selected article was not a letter, a positive value was assigned.
- Outcome: if the selected article was not a virome study, a positive value was assigned.
- Co-infections: if the selected article did not report samples with co-infections, a positive value was assigned.

The sum of the assigned points was used to stratify the selected studies as follows:

- 5 points: High
- 3–4 points: Moderate
- 1–2 points: Low

### 2.12. Additional Analyses

For the virome studies, it was possible to do a qualitative description only, which was due to the absence of the clear number of cases and the controls infected with a given virus. Thus, the viral prevalence is provided for these cohorts as a whole.

### 2.13. Statistical Analysis

The meta-analysis was implement in *R* version 4.2.1 using the packages *metafor*, *meta*, and *metasens*, which provided both the statistical and graphical outputs.

## 3. Results

### 3.1. Study Selection

**Figure 1:**
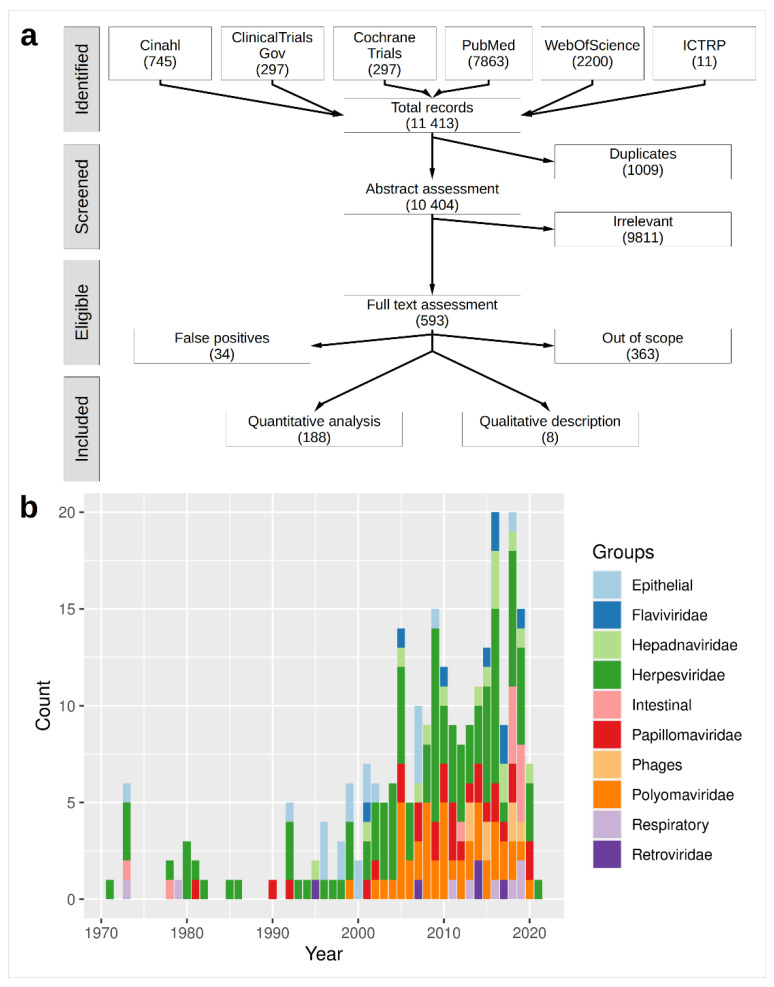
Overview of the present meta-analysis. Study selection was based on the PRISMA standard. (**a**) Flow diagram of the present meta-analysis. (**b**) Stratification of the studies by publication year and virus type.

We inquired six scientific literature databases: PubMed, Cochrane Library, Web of Science, Cinahl, ClinicalTrial.gov, and the International Clinical Trials Registry Platform (ICTRP) on 3 March 2021 and identified 11,413 articles, with 1009 duplicated entries, matching our search algorithm (Figure 1a). Following the screening for titles and abstracts, we withdrew 9811 articles because they were not related to the presence of viruses in the tissues derived from either CRC or IBD patients. An additional 364 articles were removed after reading the full text because they were not related to the research question and 34 were withdrawn because they were related to anal cancer (false positives). We identified 196 articles that matched the research question (Supplementary Table S1). We subdivided the selected articles into two groups: one undergoing a quantitative analysis (n = 188, 95.9%) and another qualitative description (n = 8, 4.1%). This subdivision was due to the fact that the latter group comprised a MPS-derived virome analysis not reporting the number of patients infected with a given virus, but rather presenting an overview of the viral families observed in the whole sample set. It was therefore not possible to calculate the ORs for these studies but they were included because they represented the most recent advancements in CRC research and highlighted the presence of viruses that could not be determined by more conventional methods.

### 3.2. Study Characteristics

Since there were 38 articles of the 196 studies retrieved (19.4%) which reported results for more than one virus, there were collectively 255 data points, 211 of them (82.7%) performed after the year 2000 (Figure 1b). There were 117 data points out of 196 (45.9%) that did not provide a comparison group; thus, these articles were not suitable for the quantitative assessment.

The data points could be stratified, according to the following groups:

#### 3.2.1. Disease

- CRC (n = 123, 48.2%).
- GC (n = 28, 11.0%).
- IBD (n = 104, 40.8%).

#### 3.2.2. Detection Method

- *Hybridization* (n = 49, 18.8%). Fluorescent immuno-assay (IFA), in situ hybridization (ISH) or immuno-histochemistry (IHC).
- *PCR* (n = 118, 46.5%). Polymerase chain reaction (PCR), both end-point or quantitative.
- *Archival* (n = 25, 9.8%). Medical records obtained from the retrospective studies, and without direct detection of the virus within the specimens.
- *Serology* (n = 55, 21.6%). Enzyme-linked immunoassay (ELISA) and plaque assay.
- *Virome* (n = 9, 3.5%). Whole-genome sequencing (WGS) by massively parallel sequencing (MPS).

#### 3.2.3. Type of Tissue

- Colon and rectum tissue (n = 114, 44.7%).
- Gastrointestinal (stomach) tissue (n = 43, 16.9%).
- Stools (n = 18, 7.1%).
- Sera (n = 56, 22.0%).
- Medical records (n = 24, 9.4%).

#### 3.2.4. Viral Groups

- Respiratory viruses (n = 8, 3.1%).
- Intestinal viruses (n = 11, 4.3%).
- Epithelial viruses (n = 20, 7.8%).
- Herpesviruses (n = 107, 42.0%).
- Retroviruses (n = 5, 2.0%).
- Polyomaviruses (n = 44, 17.3%).
- Papillomaviruses (n = 28, 11.0%).
- Hepadnaviruses (n = 16, 6.3%).
- Flaviviruses (n = 9, 3.5%).
- Phage group (n = 7, 2.7%).

The majority of the 196 studies displayed a moderate NOS score (n = 127, 64.8%), followed by a high NOS score (n = 60, 30.6%), and only a fraction of the studies retrieved herein had a low score (n = 9, 4.6%). Therefore, it could be assumed that the quality of the dataset was good (Supplementary Table S2).

### 3.3. Quantitative Analysis: CRC

The risk of developing either CRC, GC or IBD due to a viral infection based on the random effects model, is reported on Table 1. Based on the colorectal tissues, there was a significant association between an infection and CRC for the viral groups herpes (OR = 2.62, 95% CI: 1.17–5.86, *p*-value = 0.0187), Polyomaviruses (OR = 2.17, 95% CI: 1.20–3.93, *p*-value = 0.0105), Hepadnaviruses (OR = 1.22, 95% CI: 1.16–1.28, *p*-value < 0.0001), and epithelial viruses (OR = 3.76, 95% CI: 1.21–11.68, *p*-value = 0.0221). An infection with Papillomaviruses was just above significance (OR = 4.56, 95% CI: 0.99–21.03, *p*-value = 0.0512). Based on the sera, none of the studies indicated a significant increase of risk of a gastrointestinal disease. Conversely, based on the archival records, the random effects model identified a significant association between infection with Flaviviruses (OR = 0.63, 95% CI: 0.04–0.10, *p*-value < 0.0001) and confirmed the association between an infection with Hepadnaviruses and CRC (OR = 0.12, 95% CI: 0.08–0.17, *p*-value < 0.0001). However, the results obtained from the archival records was based on individual studies and pointed to a decreased risk of cancer upon infection. None of the indices related to the quantification of study heterogeneity (*I²* index, ERT, and CQT) could be computed, confirming the low reliability of the results, based on archival data.

Similarly, the results regarding the increased risk of CRC upon infection with HBV, based on the colorectal tissues, was based on a single study and even in this case no measure of heterogeneity could be computed. The association between the increased rick of CRC and infection with epithelial viruses was slightly better because, based on two studies that consistently reported a higher cancer risk upon infection, only the CQT could be computed (*p*-value = 0.1803), suggesting a negligible publication bias.

**Figure 2:**
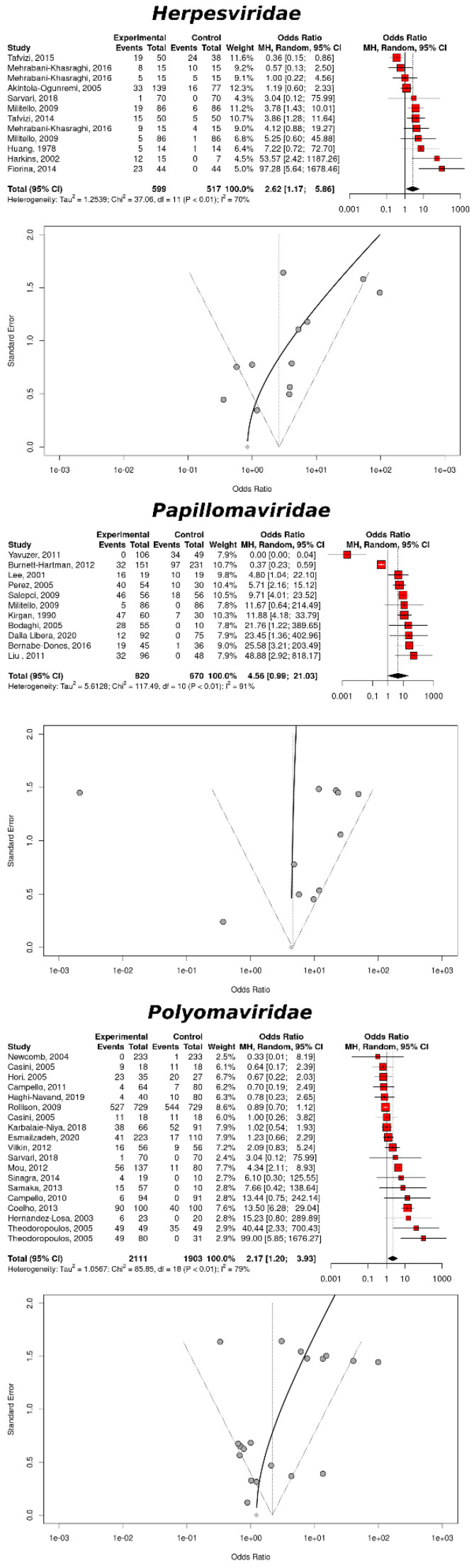
Association between the CRC risk and a viral infection. The upper, middle, and lower panels are related to the infection with members of the *Herpesviruses*, *Papillomaviridae*, and *Polyomaviridae* families, respectively, for the colorectal tissues. Each panel includes a forest plot, based the random effects models and a funnel plot with a regression line.

The relation, based on the colorectal tissues and infection with either Herpesviruses and Polyomaviruses, was better because it was based on 12 and 19 studies, respectively. In both cases, the *I²* index was considerable (≥70.3%), indicating an issue of publication bias and corroborating the use of the random effects model for computing the meta-analysis because it is more reliable in the presence of such a bias. The ERT was slightly below significance, strengthening the issue of publication bias. The observation of the funnel plots for Herpesviruses, Papillomaviruses, and Polyomaviruses, showed that the reports related to an infection with HPV were seriously affected by an asymmetric distribution (Figure 2). The situation for Herpesviruses and Polyomaviruses was better than that of Papillomaviruses, but it was still possible to observe a higher representation of the studies reporting a positive association between an infection and CRC than the studies describing otherwise.

Given the influence of the publication bias in defining the association between viral infection and CRC, the trim and fill method was applied (Table 2). The ERT became significant for Herpesviruses, Papillomaviruses, and Polyomaviruses (*p*-value < 0.0001 in all cases), whereas the CQT became non-significant. Given the small sample set, the former metric should be considered more reliable. However, none of the viruses was associated with a significant risk of CRC; thus, the increased risk of CRC, based upon an infection with either Herpesviruses and Polyomaviruses, could not be confirmed by the trim-and-fill method.

Further stratification of the CRC risk, based on a Herpesviruses infection, was based on the viral genera of the *Herpesviridae* family (Table 3). Based on the colorectal tissues, HHV-4 showed an increased CRC risk (OR = 3.39, 95% CI: 0.64–18.10), but with a non-significant association (*p*-value = 0.1531). HHV-5 was instead just above significance (OR = 2.71, 95% CI: 0.99–7.45, *p*-value = 0.0519). HHV-1 (Herpes Simplex 1) and HHV-8 (Kaposi sarcoma herpes virus), with only one study each, did not provide valuable data to the analysis.

**Figure 3:**
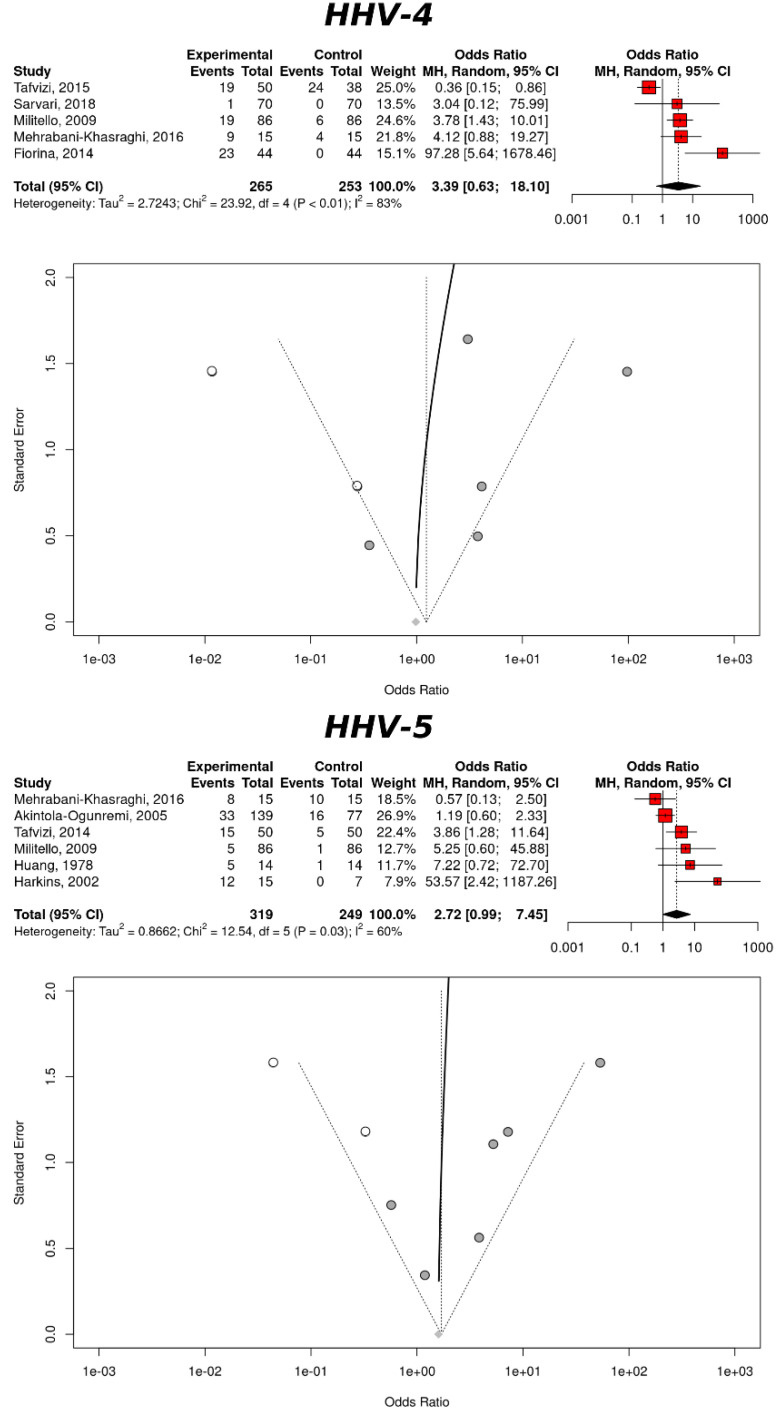
CRC risk and an infection with Herpesviruses, based on the colorectal tissues. The upper panel refers to HHV-4 (Epstein–Barr virus) and the lower panel refer to HHV-5 (Cytomegalovirus). Each panel includes a forest plot, based the random effects models and a funnel plot with a regression line. The studies added by the trim-and-fill method are represented by white dots.

The heterogeneity associated with the studies related to HHV-4, was considerable (*I*² = 83.3%) but moderate for HHV-5 (*I*² = 60.1%), albeit the CQT was significant in both cases. The ERT could not be calculated for either sub-group. The application of the trim-and-fill method resulted in the addition of two studies with both HHV-4 and HHV-5. For the former, the association produced the values: OR = 1.24, 95% CI: 0.19–8.21, *p*-value = 0.8268, *I*² = 84.3% (95% CI: 69.3–91.9%), CQT < 0.0001; for the latter the values were: OR = 1.70, 95% CI: 0.55–5.25, *p*-value = 0.3573, *I*² = 64.5% (95% CI: 24.3–83.4%), CQT = 0.0062. The ERT could not be computed for either sub-group. Therefore, even in this occurrence, the fill-and-trim method did not substantiate the results of the random effects model. A visual inspection of the data (Figure 3) confirmed a publication bias due to the over-representation of the studies reporting a positive association between an infection and the CRC risk.

The vast majority of the studies related to the CRC risk and an infection with members of the *Polyomaviridae* family (n = 36) was due to JCV (n = 27, 75.0%). The other viruses involved in the infection model were BKV (n = 4, 11.1%), SV40 (n = 3, 8.3%), and MCPV (n = 2, 5.6%). Further analysis was focused on JCV because it was the most prevalent in the study set. The random effects model reported a significant association, based on 15 studies, between an infection with JCV and the CRC risk: OR = 2.64, 95% CI: 1.27–5.49, *p*-value = 0.0096, *I*² = 82.9% (95% CI: 73.0–89.1%), ERT = 0.0524, CQT < 0.0001. However, the trim-and-fill method, which added five studies to the set, did not confirm the association: OR = 1.19, 95% CI: 0.46–3.09, *p*-value = 0.7180, *I²* = 87.3% (95% CI: 81.8–91.2%), ERT = 0.7247, CQT < 0.0001.

The epithelial group associated with a high risk of CRC, was due to an infection with TTV (n = 2) and B19 (n = 1). Due to the paucity of data, it was not possible to further stratify the analysis. The other sources of information (gastric tissues, sera, and medical records) did not provide additional value to the analysis of the CRC risk.

### 3.4. Quantitative Analysis: GC

The number of studies regarding GC was very limited. Based on the gastric tissues, only Herpesviruses and Polyomaviruses had enough coverage to provide metrics for the meta-analysis (Table 2). However, only one study out of 22 could provide information regarding Herpesviruses (OR = 1.57, 95% CI: 0.60–4.06, *p*-value = 0.3567), resulting in a non-significant association and the absence of heterogeneity measures. Only two studies provided information regarding the GC risk upon infection with Polyomaviruses, but in this case with a significant association: OR = 5.22, 95% CI: 1.66–16.47, *p*-value = 0.0048, ERT = 0). It was not possible to perform the trim-and-fill method.

Serum samples provided information for Herpesviruses and HBV, but none of the associations was significant (Herpesviruses: OR = 0.49, 95% CI: 0.13–1.78, *p*-value = 0.2774; Hepadnaviruses: OR = 1.36, 95% CI: 0.72–2.57, *p*-value = 0.3375.

### 3.5. Quantitative Analysis: IBD

Based on the colorectal tissues, the only viruses providing information to compute in the meta-analysis, were the Herpesviruses, which, however, showed an association just above significance: OR = 2.71, 95% CI: 0.91–8.06, *p*-value = 0.0723, *I²* = 50.6% (95% CI: 0–76.9%), CQT = 0.3980. The trim-and-fill method did not add any study to the set, thus it confirmed the results of the random effects model. The moderate *I²* score and the non-significant CQT, highlighted that in this case, there was a negligible publication bias and, therefore, no requirement for an adjustment of the results.

Based on stool specimens, intestinal and respiratory viruses showed a significant association with the IBD risk. There were five studies substantiating the meta-analysis, however the trend reported was that of a reduction of IBD upon infection with intestinal viruses: OR = 0.27, 95% CI: 0.09–0.08, *p*-value = 0.0236. However, the heterogeneity was substantial (*I*² = 92.0, 95% CI: 84.4–95.6, CQT < 0.0001), suggesting that there was a strong publication bias. The trim-and-fill method added three articles to the set and did not confirm the association: OR = 0.69, CI: 0.19–2.45, *p*-value = 0.5630, *I*² = 94.2% (95% CI: 90.8–96.4%), CQT < 0.0001. Remarkably, the adjustment of the data showed that the IBD risk could be as high as 2.45 upon infection, suggesting that the possible beneficial role of an infection with intestinal viruses (Astrovirus, Norovirus, Rotavirus, and Sapovirus) was most probably a statistical artifact. With only one study supporting the association between an infection with respiratory viruses (Adenovirus) and the IBD risk, the association was even weaker than for intestinal viruses: OR = 0.29, CI: 0.07–1.16, *p*-value = 0.5630. Neither the measurement of the study heterogeneity nor the trim-and-fill method could be computed.

**Figure 4:**
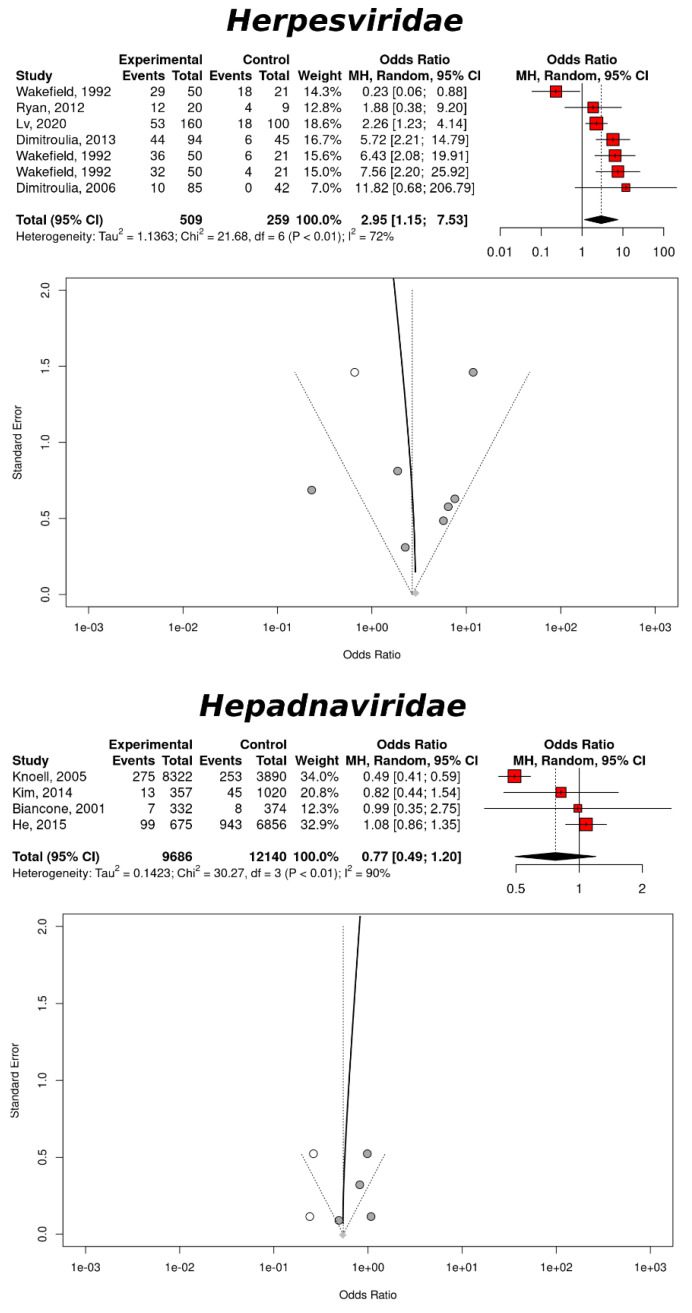
IBD risk and a viral infection. The upper panel refers to Herpesviruses, based on the gastric tissues, and the lower panel refers to Hepadnaviruses on the sera. Each panel includes a forest plot, based the random effects models and a funnel plot with a regression line. The studies added by the trim-and-fill method are represented by white dots.

Based on gastric tissues, there was a significant involvement of Herpesviruses in increasing the IBD risk. The association was supported by seven studies and provided the following measures: OR = 2.90, 95% CI: 1.15–7.53, *p*-value = 0.0241, *I*² = 72.3% (95% CI: 40.2–87.2%), CQT = 0.0014. The trim-and-fill method added one study and confirmed the association between an infection with Herpesviruses and the high risk of IBD: OR = 2.67, 95% CI: 1.10–6.52, *p*-value = 0.0307, *I*² = 69.1% (95% CI: 35.6–85.2%), CQT = 0.0019. Figure 4 shows how the trim-and-fill method improve upon the publication bias associated with these measures.

Based on the sera, there was a significant association between a high IBD risk and an infection with either respiratory viruses and Polyomaviruses. The former association was, however, based on a single study: OR = 0.38, 95% CI: 0.16–0.91, *p*-value = 0.0291. Neither the measurement of the study heterogeneity nor the trim-and-fill method could be computed. As in the case of the association between an infection with respiratory viruses and the IBD risk based on stools, the reduced risk upon infection might simply be a statistical artifact. Similarly, the association between an infection with Polyomaviruses and the IBD risk, was based on a single study: OR = 9.47, 95% CI: 3.46–25.92, *p*-value < 0.0001. In this case, there was an elevated risk but the paucity of the study set made the association untrustful. Neither the measurement of the study heterogeneity nor the trim-and-fill method could be computed.

Interestingly, there was an association between an infection with Hepadnaviruses and the IBD risk. The random effects method did not find a significant association, based on four studies related to serum specimens: OR = 0.77, 95% CI: 0.49–1.20, *p*-value = 0.2492, *I*² = 90.1% (95% CI: 77.6–95.6%), CQT < 0.0001. However, the trim-and-fill method added two studies and reported a significant association: OR = 0.55, 95% CI: 0.32–0.94, *p*-value = 0.0275, *I*² = 94.5% (95% CI: 90.6–96.8%), CQT < 0.0001 (Figure 4). The trim-and-fill method improved upon the publication bias associated with these measures. As in the case of intestinal and respiratory viruses, the reduced IBD risk upon infection might simply highlight a statistical artifact.

**Figure 5:**
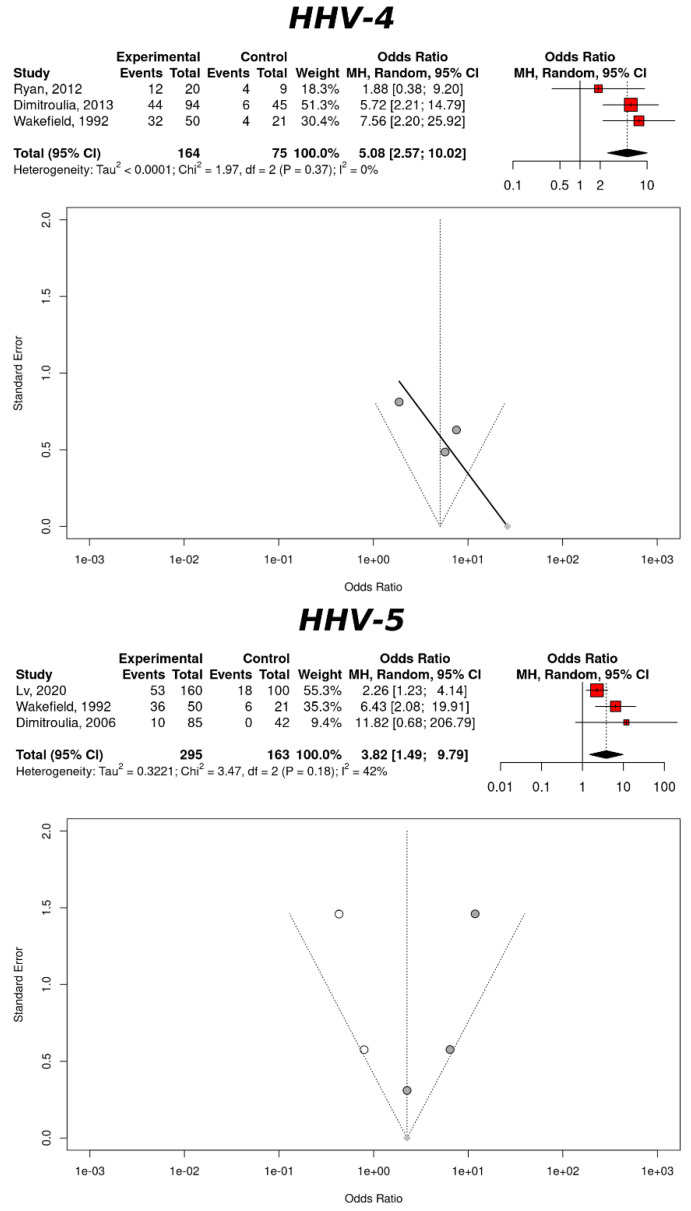
IBD risk and an infection with Herpesviruses based on the gastric tissues. The upper panel refers to HHV-4 (Epstein–Barr virus) and the lower panel refers to HHV-5 (Cytomegalovirus). Each panel includes a forest plot based on the random effects models and a funnel plot with a regression line. The studies added by the trim-and-fill method are represented by white dots.

Further stratification of the IBD risk based on a Herpesviruses infection was based on the viral genera of the *Herpesviridae* family (Table 2). Based on three studies related to the gastric tissues, HHV-4 showed a significant increase in the IBD risk: OR = 5.08, 95% CI: 2.57–10.02, *p*-value < 0.0001. The measure of heterogeneity indicated a negligible publication bias, further substantiating these results: *I*² = 0% (95% CI: 0–89.6%), CQT = 0.3740. The trim-and-fill method did not add any study, confirming the negligible publication bias associated with these measures (Figure 5). Based on three studies related to the gastric tissues, HHV-5 also showed a significant increase of the IBD risk: OR = 3.82, 95% CI: 1.49–9.79, *p*-value = 0.0053. As in the case of HHV-4, the measure of heterogeneity advocated for a low publication bias: *I*² = 42.4% (95% CI: 0–82.6%), CQT = 0.1761. The trim-and-fill method added two studies but the association was reduced to just above significance: OR = 2.26, 95% CI: 0.88–5.82, *p*-value = 0.0920, *I*² = 56.4% (95% CI: 0–83.8%), CQT = 0.0571 (Figure 5). There was only one study reporting about the IBD risk upon infection with HHV-6; the association was significant: OR = 0.23, 95% CI: 0.06–0.88, *p*-value = 0.0323. Neither the measurement of the study heterogeneity nor the trim-and-fill method could be computed.

### 3.6. Qualitative Analysis of the Virome Studies

The majority of the virome studies were based upon stool samples: 8 out of 11 (72.7%). The microbiome analysis showed a higher viral richness in the controls (n = 28) than the CD patients (n = 31): there were 4399 viral species in the former group and 2161 viral species in the latter [48,49]. *Synechococcus* phage S CBS1 was characteristically observed only in inflammatory lesions [48]. Other studies reported a higher abundance (in terms of the total number of sequences) of phages, specifically those belonging to the order *Caudovirales*, in the inflammatory lesions (n = 12) than in the healthy controls (n = 12) (Dunn’s test, *p*-value = 0.05); however, such an increase did not correspond to a rise in the phage richness [50]. An increased phage richness (specifically of the members of the *Caudovirales* family), together with a lower bacterial richness in the IBD patients (n = 52) over the healthy controls (n = 21), was instead reported by Norman and co-workers (Spearman correlation, *p*-value < 0.05) [38]. In particular, the authors identified the phages targeting the bacterial species *Lactococcus*, *Lactobacillus*, *Clostridium*, *Enterococcus* and *Streptococcus*, as specifically associated with this disease.

The comparison of the viral communities between the CRC cases (n = 74) and the healthy controls (n = 92) in the stools, showed an increased phage richness in the former (Wilcoxon rank test, *p*-value = 0.013) as well as an inverse association between the bacterial and viral richness [51]. Using the random forest analysis, the authors identified *Orthobunyavirus* as a taxon specifically associated with CRC specimens, followed by Inovirus and Tunalikevirus. Using a similar approach, Hannigan and collaborators [52] identified members of the viral species *Siphoviridae* and *Myoviridae* (both belonging to the order *Caudovirales*) as more associated with CRC (n = 60) than the healthy condition (n = 30) (Wilcoxon rank test, *p*-value < 0.01).

In one study based on colon biopsies (six CD patients and six healthy controls), the phages B40-8 and B124-14, both infecting *Bacteroidetes fragilis*, were identified as the most abundant; furthermore, the *Mycobacterium* phages TM4 and Wee were observed only in the inflammatory lesions [53]. In one study based upon the archival data, a high variability of virome communities between people was observed, although members of the *Partiviridae* and *Hepeviridae* families were the most prevalent [54]. Healthy specimens showed a higher predominance of the viral families *Polydnaviridae*, *Tymoviridae*, and *Virgaviridae* together with a significantly lower representation of *Hepeviridae*, compared to the inflammatory lesions (one-way ANOVA with Bonferroni correction, *p*-value < 0.05).

The work by Zapatka and collaborators was based on the re-analysis of the stored WGS-data, derived from 5354 pairs of tumors and normal tissues [55]. The tumors included 38 cancer types from 356 patients. The results showed that HHV-4 was the most common viral entity in the dataset, with a higher prevalence in the tumor tissues. The study also included 1057 RNA expression samples that indicated how GC sections were enriched in lytic HHV-4 transcripts, supporting a role of this virus in the gastric oncogenesis. The study did not report an infiltration of lymphocytes that could explain the presence of HHV-4 in the tumor tissues.

## 4. Discussion

Gastrointestinal diseases represent a primary public health concern. In particular, CRC and GC are highly prevalent cancer types with a high mortality and delayed diagnosis. IBD is a significant cause of morbidity that has been pointed out as a risk factor for the development of CRC. The prevalence of CRC and GC is still increasing. A genetic predisposition, dietary habits, and sedentary lifestyle, amongst other causes, are associated with developing these gastrointestinal diseases. Bacterial infections have been recognized as additional risk factors as well. However, the role of viral infections in the development of CRC, GC, and IBD is less well understood. Our present meta-analysis suggests a role for HHV-4 (EBV) as a risk factor for IBD and also, less significantly, for CRC, and of HHV-5 (CMV) as a less strong risk factor for CRC and IBD.

The present study sought to assess the role of viruses as risk factors for these main gastrointestinal diseases. This aim was fulfilled by applying a meta-analysis to the scientific publications related to the prevalence of viruses in people with CRC, GC, or IBD, against control subjects. We included all viruses associated with human diseases, including bacteriophages, to avoid the risk of missing possible links.

The most striking finding provided by the present study is the amount of literature bias on this subject. Among the causes of study heterogeneity, there might be, e.g., geographical variations or the use of disparate investigative methods [55]. In the vast majority of cases, the ERT and CQT were significant. In many cases, the groups contained too few studies to quantify heterogeneity, and heterogeneity of the studies was classified as significant in seven out of 17 studies (41.2%). Such a high study heterogeneity confirmed that the random effect model was correctly chosen for the data analysis. However, the presence of a publication bias also hampered the correct quantification of the association between a viral infection and the risk of gastrointestinal disease. Such a problem was reflected, for instance, in the possible role of HBV and HPV, in the development of CRC, based on the colorectal tissues: the former had a highly significant association but only one publication supported it, and the latter had an association just above significance, but was substantiated by 11 publications. The trim-and-fill method was used to account for the publication bias in this meta-analysis, but it could only be computed in 10 cases out of 137 (7.3%). Moreover, the employment of the trim-and-fill method did not confirm the results obtained with the random effect model, creating a contrast that made it more challenging to interpret the results. Moreover, the measures that were significant and using both methods, increase the reliability of the risks found between the particular infections and gastrointestinal diseases.

The data presented by our meta-analysis confirm the role of the particular viral infections as risk factors for CRC and IBD. The primary finding of the current meta-analysis was that, using both the random effect model and the trim-and-fill model, the OR of an infection over the absence of an infection was significantly greater than the unity, indicating that the Herpesvirus HHV-4 (EBV) was a significant risk factor in the development of IBD. The present study’s second finding was less accurate than the primary result, since it was based on the random effect model only. Still, it suggests HHV-5 as a risk factor for both CRC and IBD. The meta-analysis supported the tertiary findings that were not only based on a single model, but also hampered by a small sample set and, consequently, a high publication bias. Such results suggested that: (a) Polyomaviruses could be associated with a higher risk of both CRC and IBD; (b) HBV could be associated with a higher risk of CRC; (c) Papillomaviruses could not be ruled out as CRC risk factors since the association was just slightly above significance; and (d) respiratory, intestinal, and epithelial viruses had a very limited prognostic value on the risk of these. In contrast, there was not sufficient evidence to provide an association between a viral infection and the GC risk, due to the absence of a high enough number of strong studies.

HHV-4 (EBV) is typically associated with lymphomas, rather than gastrointestinal diseases, whereas HHV-5 is believed to cause chronic inflammation and immune dysfunction in immune-compromised people [56,57]. However, a recent MPS-based survey of tumoral tissue, demonstrated the presence of *Herpesviridae* in colorectal (HHV-4), gastric (HHV-4 and HHV-5) and liver (HHV-4, HHV-5) tissues [58]. These findings were confirmed by our own recent metagenomic analysis of CRC and the CRC metastasis tissues, which identified the presence of both HHV-4 and HHV-5 [59]. Remarkably, further recent studies showed a concomitant presence of HPV and HHV-4 in CRC. In a group of 102 CRC patients, 17% showed a concomitant presence of HPV and HHV-4, with a higher risk of tumor progression compared to patients infected with only one virus type [60], suggesting that HPV and EBV might interact synergistically, fostering the development of several types of cancer, including CRC [61]. In this context, it is established that the immune suppression triggers the reactivation of the latent viruses, such as HHV-5 [62,63].

It is well known that HHV-4 can disrupt the cell cycle at several points, fostering the genesis of cancer and even metastasis [61,64]. For instance, the EBV-derived LMP1 disrupts several cellular signal pathways (such as NF-κB, PI_3_K, and MAPK) [65] and reduces the expression of E-cadherin [66], which is also targeted by HHV-4′s micro interfering RNAs, such as miR-BART9 [67]. LMP2A is associated with increased metastasis [68]. Additionally, HHV-4 interacts with HER-2, fostering an increased metastatic capacity [69] and the EBV-associated EBNA-1 is usually over-expressed in metastases [70]. Based on these observations, it has been suggested that HPV and HHV-4 might interact synergically, increasing the risk for carcinogenesis and metastasis. Towards this end, HHV-4 would act in the first steps of the cellular transformation, and HPV would continue to induce metastatic abilities [71]. The oncogenic mechanism of action of HHV-5 is still not completely understood, but it has been shown that an infection with this virus increases the viability and migration of the CRC-derived cell lines [72].

HHV-4 (EBV) has been shown to be more prevalent in IBD patients [73,74]. For instance, in one study, the prevalence of HHV-4 in IBD patients was 33%, as compared to 7% in the healthy controls [75]. Moreover, the presence of HHV-4 in the intestinal tissues is known to exacerbate the symptoms associated with IBD [76,77]. It is assumed that HHV-4 infects the T cells rather than the epithelial cells, the T-cells then infiltrate the intestinal tissues; thus, the inflamed tissues have a higher probability of carrying HHV-4 genomes [78]. In analyzing the CRC tissues for the HHV-4 presence, using in situ hybridization, our group observed signals consistent with the infection of lymphocytes rather than colonocytes, indeed [79]. Still, the detection of HHV-4 in tissues is tricky and requires ISH approaches that are not routinely performed [78]. Similarly, the detection of HHV-5 has not been standardized [80], increasing the discrepancies in the viral prevalence between the studies. This might explain why Herpesviruses are still not consistently observed in gastric or colorectal tissues, and/or in different frequencies or quality.

IBD is a CRC precursor [81]. Since IBD is treated with immunosuppression (for instance, azathioprine and infliximab) and EBV fosters in the absence of a fully functional immune system, the risk of viral-driven oncogenesis might be increased [82,83]. The reactivation of latent HHV-4 infections has been shown to cause lymphomas in IBD patients treated with azathioprine [84]. Moreover, the immunosuppression that results from the therapy of IBD, stimulates HHV-4 replication, provoking more inflammation [74,85]. This not only explains why IBD treatment is sometimes ineffective, due to the recurrence of inflammation, but also clarifies why the risk of developing lymphomas in IBD patients under immunosuppressive therapy, is high [86].

Therapy with immune suppressants, such as infliximab, is also involved in the reactivation of Hepadnaviruses [87]. The main member of this family, HBV, is the causative agent of hepatocellular carcinoma, rather than GC or CRC [88]. HBV causes about 1.5 million deaths yearly and IBD patients are at an increased risk of infection [89], as the present study has also confirmed. The X protein encoded by this virus is understood to disrupt the expression of several cellular genes involved in apoptosis, DNA repair, oxidative stress control, and immune response [90]. Remarkably, a HBV infection is treated routinely with immune suppressants. The reactivation of HBV, due to immunosuppression might have deleterious consequences for the patients. For instance, it has been reported that the high HBV serum titers are associated with a higher risk of metastasis in CRC [91]. Therefore, it is plausible to hypothesize that the use of immune suppressants in IBD patients with a latent HBV infection, might reactivate the virus, causing, as in the case of EBV, the instauration of chronic inflammation that will neutralize the effectiveness of the therapy and, in turn, foster the development of CRC. Consequently, it is recommended to screen IBD patients for the presence of a HBV infection [92].

As in the case of Herpesviruses and Hepadnaviruses, Polyomaviruses can also be reactivated by immunosuppressive therapy [93,94,95]. It has been suggested that latent JCV might be reactivated in cases of IBD [96]. JCV can cause IBD by altering the immune system [97]. Similarly, Papillomaviruses are not typically associated with gastrointestinal diseases. Papillomaviruses are observed in over 36% of cervical and oropharyngeal cancers, in about 12% of anal cancers, but in quite variable frequencies in CRCs, for example less than 2% in one study [98]. In contrast, Kirgan and collaborators reported a viral prevalence of 97% in the CRC tissues and 23% in matched normal colon mucosa for HPV, with a significant association between a HPV infection and the CRC risk [99]. Based on such heterogeneous data, other meta-analyses confirmed a Papillomavirus prevalence of 42–83% in the CRC tissues [100,101,102,103], and reported ORs of 5-10 for the CRC risk upon infection with HPV [104,105], which is in line with the value of the 4.56 (95% CI: 0.99–21.03) we observed in our study.

About a decade ago, HHV-4 was described as a causative agent of the Epstein–Barr virus-positive mucocutaneous ulcer (EBVMCU), a condition affecting the pharyngeal and gastrointestinal mucosa, with ulcers refractory to treatment and displaying histological features typical of HHV-4 associated lymphomas [106,107]. EBVMCU has also been reported in the colon and rectum [108] as has been a lymphoma of the colon, symptomatically resembling IBD, caused by HHV-4 [109]. A HHV-4 infection of the gastrointestinal tract might trigger symptoms resembling IBD, leading to mistreatment that actually fosters the establishment of IBD [110]. In general, it is becoming clear that a HHV-4 infection of the gastrointestinal tract might not only make it difficult to distinguish it from IBD, but also increases the risk of mistreatment, treatment resistance, and cancer [111,112].

Taken together, these findings suggest that intestinal damage can be caused by viruses that induce chronic inflammation without directly residing in the tissues of the gastrointestinal tract. HHV-4 might reside in the immune cells, HHV-5 in several tissues, and HBV in the liver, still able to affect the gastrointestinal tract by causing a generalized chronic inflammation and aberrant immune response. This framework might explain why these viruses are not consistently found in CRC and IBD cases. Another explanation for the lack of a clear relationship between a viral infection and CRC, might be that viruses can cause ‘hit-and-run’ mutagenesis [113,114]. Remarkably, ‘hit-and-run’ instances have been described for both Herpesviruses and Papillomaviruses [115]. Mouse models have demonstrated how HHV-4 can cause lymphomas, but then the virus is lost, making virus detection difficult. This emphasizes the need to shift the focus of virus research from the positivity of cancer tissues and cells [116]. It is possible to imagine viruses infecting colonocytes, pushing the infected cells to transformation, but then being cleared from the tissue. In addition, the virus-caused chronic inflammation per se might foster carcinomas. This might be achieved not only by known viruses, but of course by yet not well described species which we could not analyze in our present meta-analysis, due to the fact that they are not yet annotated sufficiently (e.g., [35]).

The present review included the analysis of the MPS-derived studies as well. These, being qualitative in essence, could not be used for the quantification of the risk of gastrointestinal diseases upon infection. Although MPS-based studies provide a broader overview of the presence of microbes in samples, they are, at the moment, not suitable for quantitative analysis because they are not designed to calculate ORs. The statistical methods applied in these studies are designed to indicate trends in the presence of microbes in the samples, but not to reveal a measured risk associated with a disease. Nevertheless, the MPS-based studies, including our own, consistently reported a higher viral richness (the number of species) in the inflamed or tumor tissues [117]. A weakened immune system might trigger the reactivation of latent viruses already present in a tissue, increasing their viral load over the detection limit. In addition, inflammation might cause tissue damage [118,119] that can facilitate the infection of tissues and the expansion of viruses.

Conversely, MPS-derived studies including our recent one, reported a decreased bacterial richness as well [38,51,117]. This feature raises an apparent contradiction: if a dysfunctional immune system can foster a viral infection, why would it not do the same for bacteria? It might be speculated that, on the one hand, the reduction in bacterial species might be due to the reduction of commensal species, which do not thrive in inflamed tissues. On the other hand, the result of a viral expansion includes bacteriophages; thus, it is plausible that a higher proportion of bacteria might be lysed in an inflamed tissue. Although our understanding of the dynamics underlying intestinal microbiota is still poor, it is assumed that the gut microbiome is enriched in lysogenic viruses [120]. Thus, it is interesting to hypothesize that a significant rise of phage quantity might occur in the inflamed tissues, including the tumor regions, reducing the amount, and maybe diversity, of the bacteria.

The present study had some limitations. First, the present meta-analysis considered each viral group as a whole regardless of the detection method employed. However, such heterogeneity might have introduced a bias in the results. Ideally, the prevalence obtained with one technique should be confirmed by another method, but this is rarely the case. Herein, the subdivision of the results by detection method, would have reduced the number of studies in each class, hampering their statistical evaluation. The present study aimed to identify the possible trends in the association between a viral infection and intestinal disease that should be further investigated at the experimental level. Second, it was also impossible to include every type of virus. Despite using the general term “virus” as a keyword during the literature search, viruses known to cause gastroenteritis during the acute phase of infection, such as the *Orthomyxoviridae* family, did not appear in the results. While a comprehensive review of the relationship between all types of viruses and intestinal diseases may never be complete, the lack of reference between an infection with viruses, such as orthomyxoviruses and CRC, GC, or IBD, suggests that the link is tenuous. Third, the lack of longitudinal data made it impossible to determine whether the infection was a cause or result of intestinal diseases. The presented study only reported a statistical association between an infection and disease; more detailed studies will be required to determine the causality comprehensively.

## 5. Conclusions

The present study investigated the prevalence of viruses by a meta-analysis of the actual status of relevant scientific publications in CRC, GC, and IBD. The meta-analysis highlights a significant publication bias in the literature related to this topic. Taking this into account, the present study suggests that HHV-4 (EBV) is a significant risk factor in the development of IBD, and points to HHV-5 as a risk factor for both CRC and IBD. Furthermore, the meta-analysis suggests that, to a less significant extent, Polyomaviruses might play a role in the risk of both CRC and IBD, and that HBV might be a risk factor for CRC.

## Figures and Tables

**Table 1:** Mixed-affect model for the risk of CRC, GC, and IBD predicted by the studies selected for the present meta-analysis. The studies are stratified by specimen type (Source) and virus type (Group). The ORs are reported with their 95% confidence interval and *p*-value. *I*² = Higgins index; ERT = Egger’s regression test; CQT = Cochran’s Q test.

Disease	Source	Group	Studies *	Cases ^†^	Controls ^†^	OR	*p*-Value	*I*² (%)	ERT	CQT
CRC	Colon/Rectum	Herpes	12/28	375/1968	72/517	2.62 (1.17–5.86)	**0.0187**	70.3 (46.4–88.6)	0.0417	0.0001
		Papilloma	11/26	517/2402	177/888	4.56 (0.99–21.03)	0.0512	91.5 (86.8–94.5)	0.1270	< 0.0001
		Polyoma	19/36	1531/3980	768/2029	2.17 (1.20–3.93)	**0.0105**	79.0 (67.9–86.3)	0.0413	< 0.0001
		Hepadna	1/1	3536/69,478	2924/69,478	1.22 (1.16–1.28)	**<0.0001**	—	—	—
		Epithelial	2/3	116/132	86/119	3.76 (1.21–11.68)	**0.0221**	—	—	0.1803
		Papilloma	1/1	21/97	42/184	0.93 (0.52–1.69)	0.8221	—	—	—
		Hepadna	1/1	72/284	212/284	0.12 (0.08–0.17)	**<0.0001**	—	—	—
		Flavivirus	1/1	51/255	204/255	0.63 (0.04–0.10)	**<0.0001**	—	—	—
		Retrovirus	2/3	146/674	284/1542	0.82 (0.58–1.18)	0.2898	—	—	0.3099
	Serum	Herpes	5/5	64/89	37/79	3.06 (0.74–12.65)	0.1227	54.3 (0–83.2)	—	0.6740
		Polyoma	3/5	1029/1409	1008/1409	1.12 (0.89–1.42)	0.3224	30.9 (0–92.8)	—	0.2351
		Hepadna	1/3	674/6333	3168/96,000	1.31 (0.89–1.95)	0.1754	—	—	—
		Flavivirus	1/3	10/820	806/96,000	1.58 (0.78–3.19)	0.2009	—	—	—
GC	Gastric	Herpes	1/22	502/5097	207/225	1.57 (0.60–4.06)	0.3567	—	—	—
		Papilloma	0/1	9/302	0/0	—	—	—	—	—
		Polyoma	2/2	18/80	4/82	5.22 (1.66–16.47)	**0.0048**	0	0.6804	—
		Epithelial	0/1	17/32	0/0	—	—	—	—	—
	Serum	Herpes	1/1	43/48	88/93	0.49 (0.13–1.78)	0.2774	—	—	—
		Hepadna	1/1	26/150	20/150	1.36 (0.72–2.57)	0.3375	—	—	—
		Retrovirus	0/1	0/150	0/150	—	—	—	—	—
IBD	Colon/Rectum	Herpes	9/16	173/882	10/211	2.71 (0.91–8.06)	0.0723	50.6 (0–76.9)	—	0.3980
	Stools	Herpes	0/1	9/400	0/0	—	—	—	—	—
		Intestinal	5/7	65/2609	3108/35,321	0.27 (0.09–0.08)	**0.0236**	92.0 (84.4–95.6)	—	< 0.0001
		Respiratory	1/2	3/711	106/8826	0.29 (0.07–1.16)	0.0801	—	—	—
	Gastric	Herpes	7/10	235/591	56/259	2.90 (1.15–7.53)	**0.0241**	72.3 (40.2–87.2)	—	0.0014
		Epithelial	1/6	17/127	11/62	0.89 (0.27–2.97)	0.8530	—	—	—
	Record	Herpes	2/7	636/46,752	1083/33,355	1.22 (0.74–2.01)	0.4377	60.8 (0–90.9)	—	0.1100
		Intestinal	2/2	110/176,856	392/873,014	1.41 (0.92–2.17)	0.1174	75.7 (0–94.5)	—	0.0425
		Respiratory	1/1	6/88,428	11/436,507	2.69 (1.00–7.28)	0.0510	—	—	0.0000
		Hepadna	1/2	196/3776	4/67	0.37 (0.13–1.07)	0.0671	—	—	0.0000
		Flavivirus	1/2	34/3556	1/57	0.75 (0.10–5.64)	0.7799	—	—	0.0000
		Epithelial	2/2	38/12,738	36/15,264	1.27 (0.80–2.00)	0.3140	0	—	0.7617
		Retrovirus	1/1	2/1816	1/84	0.09 (0.01–1.02)	0.0519	—	—	0.0000
	Serum	Herpes	5/12	1926/2267	256/438	1.53 (0.52–4.46)	0.4379	82.7 (60.5–92.5)	—	0.0001
		Intestinal	2/3	62/200	23/89	1.08 (0.47–2.49)	0.8615	0	—	0.8542
		Respiratory	1/1	11/57	20/52	0.38 (0.16–0.91)	**0.0291**	—	—	0.0000
		Polyoma	1/2	158/244	6/53	9.47 (3.46–25.92)	**<0.0001**	—	—	0.0000
		Hepadna	4/8	591/10,769	1249/12,140	0.77 (0.49–1.20)	0.2492	90.1 (77.6–95.6)	—	< 0.0001
		Flavivirus	2/3	104/8969	66/4264	0.99 (0.46–2.12)	0.9734	78.2 (4.9–95.0)	—	0.0323
		Epithelial	7/8	888/1372	729/1058	0.71 (0.37–1.36)	0.3027	70.4 (35.3–86.5)	—	0.0025

* The first number in front of the division sign ‘/’ refers to the number of studies included in the statistical calculations; the second number refers to the total number of studies available for each specific combination of disease, source, and group. † The first number in front of the division sign ‘/’ refers to the number of patients infected with a given virus; the second number refers to the total number of patients available for each specific combination of disease, source, and group.

**Table 2:** Trim-and-fill model for the risk of CRC, GC, and IBD, predicted by the studies selected for the present meta-analysis. The model added a specified number of studies to adjust for the publication bias. The studies are stratified by specimen type (Source) and virus type (Group). The ORs are reported with their 95% confidence interval and *p*-value. *I*² = Higgins index; ERT = Egger’s regression test; CQT = Cochran’s Q test.

Disease	Source	Group	Added Studies	OR	*p*-Value	*I*² (%)	ERT	CQT
CRC	Colorectal tissue	Herpes	4	1.45 (0.58–3.65)	0.4284	73.2 (56.0–83.7)	<0.0001	0.7797
		Papilloma	4	1.59 (0.36–7.04)	0.5424	90.0 (85.2–93.2)	<0.0001	0.5741
		Polyoma	6	1.13 (0.52–2.44)	0.7596	84.8 (78.6–89.1)	<0.0001	0.7587
	Serum	Herpes	2	1.40 (0.26–7.69)	0.6958	66.8 (25.9–85.1)	0.0061	—
IBD	Colorectal tissue	Herpes	0	2.71 (0.91–8.06)	0.0723	50.6 (0–76.9)	—	0.398
		Intestinal	3	0.69 (0.19–2.45)	0.5630	94.2 (90.8–96.4)	—	<0.0001
	Gastric tissue	Herpes	1	2.67 (1.10–6.52)	**0.0307**	69.1 (35.6–85.2)	—	0.0019
	Serum	Herpes	1	2.08 (0.60–7.20)	0.2472	81.8 (61.3–91.5)	—	<0.0001
		Hepadna	2	0.55 (0.32–0.94)	**0.0275**	94.5 (90.6–96.8)	—	<0.0001
		Epithelial	0	0.71 (0.37–1.36)	0.3027	70.4 (35.3–86.5)	—	<0.0001

**Table 3:** Mixed-affect model for the risk of CRC, and IBD, predicted by the studies related to an infection with Herpesviruses. The studies are stratified by specimen type (Source) and Herpesvirus genotype (Group). The ORs are reported with their 95% confidence interval and *p*-value. *I*² = Higgins index; ERT = Egger’s regression test; CQT = Cochran’s Q test.

Disease	Source	Group	Studies *	Observations ^†^	Events ^†^	OR	*p*-Value	*I*² (%)	ERT	CQT
CRC	Colorectal tissue	HHV-1	1/1	5/15	5/15	1 (0.22–4.56)	1	—	—	—
		HHV-4	5/11	140/912	34/253	3.39 (0.64–18.10)	0.1531	83.3 (62.0–92.6)	—	<0.0001
		HHV-5	6/15	228/855	33/249	2.71 (0.99–7.45)	0.0519	60.1 (2.2–83.8)	—	0.0281
		HHV-8	0/1	2/186	0/0	—	—	—	—	—
IBD	Gastric tissue	HHV-4	3/4	89/195	14/75	5.08 (2.57–10.02)	**<0.0001**	0 (0–89.6)	—	0.374
		HHV-5	3/5	117/346	24/163	3.82 (1.49–9.79)	**0.0053**	42.4 (0–82.6)	—	0.1761
		HHV-6	1/1	29/50	18/21	0.23 (0.06–0.88)	**0.0323**	—	—	—

* The first number in front of the division sign ‘/’ refers to the number of studies included in the statistical calculations; the second number refers to the total number of studies available for each specific combination of disease, source, and group. † The first number in front of the division sign ‘/’ refers to the number of patients infected with a given virus; the second number refers to the total number of patients available for each specific combination of disease, source, and group.

## Data Availability

Not applicable.

## Supplementary Materials

The following supporting information can be downloaded at: www.mdpi.com/article/10.3390/cancers14205085/s1, Table S1: Studies included in the present meta-analysis, including quantitative and qualitative assessment. References [38,48,49,51,52,53,54,55,73,75,93,99,100,121,122,123,124,125,126,127,128,129,130,131,132,133,134,135,136,137,138,139,140,141,142,143,144,145,146,147,148,149,150,151,152,153,154,155,156,157,158,159,160,161,162,163,164,165,166,167,168,169,170,171,172,173,174,175,176,177,178,179,180,181,182,183,184,185,186,187,188,189,190,191,192,193,194,195,196,197,198,199,200,201,202,203,204,205,206,207,208,209,210,211,212,213,214,215,216,217,218,219,220,221,222,223,224,225,226,227,228,229,230,231,232,233,234,235,236,237,238,239,240,241,242,243,244,245,246,247,248,249,250,251,252,253,254,255,256,257,258,259,260,261,262,263,264,265,266,267,268,269,270,271,272,273,274,275,276,277,278,279,280,281,282,283,284,285,286,287,288,289,290,291,292,293,294,295,296,297,298,299,300,301,302,303]; Table S2: NOS scores of the studies included in the present meta-analysis, including quantitative and qualitative assessment.
