# Supplementary material for: Involvement of HHV-4 (Epstein–Barr Virus) and HHV-5 (Cytomegalovirus) in Inflammatory Bowel Disease and Colorectal Cancer: A Meta-Analysis
Source: Cancers (Basel). 2022 Oct 17;14(20):5085. doi: 10.3390/cancers14205085 (PMC9599759; doi:10.3390/cancers14205085)
Supplement: Supplementary file 1 [file cancers-14-05085-s001.zip › cancers-1957721-supplementary.pdf]

Table S1. Studies included in the present meta-analysis, including quantitative and qualitative assessment.

Study	Method	Source	Disease	Virus	Family	Ref.
Aarnio *et al.*, 2012	ISH	Gastric tissue	IBD	HHV-5	*Herpesviridae*	[121]
Abdel-Moneim *et al.*, 2016	PCR	Colon/rectum tissue	CRC	Bocavirus	Respiratory	[122]
Abdirad *et al.*, 2007	PCR	Gastric tissue	GC	HHV-4	*Hepadnaviridae*	[123]
Adams *et al.*, 2016	Record	—	IBD	HHV-3	*Herpesviridae*	[124]
Afzal *et al.*, 2000	PCR	Gastric tissue	IBD	Measles	Epithelial	[125]
Afzal *et al.*, 1998	Serology	Serum	IBD	Measles	Epithelial	[126]
Aghakhani *et al.*, 2014	PCR	Colon/rectum tissue	CRC	HPV	*Papillomaviridae*	[127]
Ahmad *et al.*, 2019	PCR	Stool	IBD	Norovirus, Rotavirus, ADV	Intestinal	[128]
Akintola-Ogunremi *et al.*, 2005	ISH	Colon/rectum tissue	CRC	HHV-5	*Herpesviridae*	[129]
Alacam	PCR	Gastric tissue	GC	HHV-5	*Herpesviridae*	[130]
Alain *et al.*, 2005	Serology	Serum	IBD	HHV-5	*Herpesviridae*	[131]
Alipov *et al.*, 2005	ISH	Gastric tissue	GC	HHV-4	*Herpesviridae*	[132]
Audeau *et al.*, 2002	ISH	Colon/rectum tissue	CRC	HPV	*Papillomaviridae*	[133]
Axelrad *et al.*, 2018	PCR	Stool	IBD	Norovirus, Rotavirus, ADV, Sapovirus	Intestinal	[134]
Axelrad *et al.*, 2019	Record	—	IBD	Norovirus, Rotavirus, ADV	Intestinal	[135]
Balzola *et al.*, 1998	Serology	Serum	IBD	Measles	Epithelial	[136]
Baran *et al.*, 2018	Serology	Serum	IBD	HHV-4, HHV-5	*Herpesviridae*	[137]
Bellaguarda *et al.*, 2015	Serology	Serum	IBD	JCV	*Polyomaviridae*	[138]
Bender *et al.*, 2009	PCR	Colon/rectum tissue	CRC	HHV-5	*Herpesviridae*	[139]
Bernabe-Dones *et al.*, 2016	PCR	Colon/rectum tissue	CRC	HPV	*Papillomaviridae*	[140]
Bernstein *et al.*, 2007	Serology	Serum	IBD	Measles, Mumps	Epithelial	[141]
Bertalot *et al.*, 2001	ISH	Gastric tissue	IBD	HHV-4	*Herpesviridae*	[142]
Biancone *et al.*, 2001	Serology	Serum	IBD	HBV, HCV	*Hepadnaviridae, Flaviviridae*	[143]
Bodaghi *et al.*, 2005	PCR	Colon/rectum tissue	CRC	HPV	*Papillomaviridae*	[144]
Boltin *et al.*, 2010	PCR	Gastric tissue	IBD	JCV	*Polyomaviridae*	[93]
Brichacek *et al.*, 1980	Serology	Serum	CRC	HHV-5, HHV-1, HHV-3	*Herpesviridae*	[145]
Burnett-Hartman *et al.*, 2011	Serology	Record	CRC	HPV	*Papillomaviridae*	[146]
Burnett-Hartman *et al.*, 2012	PCR	Colon/rectum tissue	CRC	HPV	*Papillomaviridae*	[147]
Burnett-Hartman *et al.*, 2013	PCR	Colon/rectum tissue	CRC	HPV	*Papillomaviridae*	[148]
Butt *et al.*, 2016	Serology	Serum	CRC	JCV, PyV6	*Polyomaviridae*	[149]

Campello *et al.*, 2010	PCR	Colon/rectum tissue	CRC	SV40, BKV	*Polyomaviridae*	[150]
Campello *et al.*, 2011	PCR	Colon/rectum tissue	CRC	MCPV	*Herpesviridae*	[151]
Cardenas-Mondragon *et al.*, 2015	Serology	Serum	GC	HHV-4	*Herpesviridae*	[152]
Carrascal *et al.*, 2003	ISH	Gastric tissue	GC	HHV-4	*Herpesviridae*	[153]
Casini *et al.*, 2005	ISH	Colon/rectum tissue	CRC	JCV, BKV	*Polyomaviridae*	[154]
Chan *et al.*, 2016	Serology	Serum	IBD	HBV	*Hepadnaviridae*	[155]
Chen *et al.*, 2016a	PCR	Colon/rectum tissue	CRC	HHV-5	*Herpesviridae*	[156]
Chen *et al.*, 2016b	PCR	Colon/rectum tissue	CRC	HHV-5	*Herpesviridae*	[157]
Chen *et al.*, 2017	Record	—	IBD	HBV, HCV	*Hepadnaviridae, Flaviviridae*	[158]
Chevaux *et al.*, 2010	Serology	Serum	IBD	HBV, HCV	*Hepadnaviridae, Flaviviridae*	[159]
Cho *et al.*, 2001	ISH	Colon/rectum tissue	CRC	HHV-4	*Herpesviridae*	[160]
Coelho *et al.*, 2013	PCR	Colon/rectum tissue	CRC	JCV	*Polyomaviridae*	[161]
Cohen *et al.*, 2018	ISH	Colon/rectum tissue	IBD	HHV-5	*Herpesviridae*	[162]
Dalla Libera *et al.*, 2020	PCR	Colon/rectum tissue	CRC	HPV	*Papillomaviridae*	[163]
Damin *et al.*, 2007	PCR	Colon/rectum tissue	CRC	HPV	*Papillomaviridae*	[100]
De Francisco *et al.*, 2018	Serology	Serum	IBD	HHV-4	*Herpesviridae*	[164]
de Lima *et al.*, 2012	PCR	Gastric tissue	GC	HHV-4	*Herpesviridae*	[165]
de Saussure *et al.*, 2004	Serology	Serum	IBD	HHV-5	*Herpesviridae*	[166]
de Souza *et al.*, 2018	ISH	Gastric tissue	GC	HHV-4, HPV	*Herpesviridae*	[167]
de Villiers *et al.*, 2002	ISH	Colon/rectum tissue	CRC	TTV	*Anelloviridae*	[168]
de Villiers *et al.*, 2007	PCR	Colon/rectum tissue	CRC	TTV	*Anelloviridae*	[169]
Del Moral-Hernandez *et al.*, 2019	PCR	Gastric tissue	GC	HHV-4, HHV-5	*Herpesviridae*	[170]
Deschoolmeester *et al.*, 2010	PCR	Colon/rectum tissue	CRC	HPV	*Papillomaviridae*	[171]
Dimitroulia *et al.*, 2006	ISH	Gastric tissue	IBD	HHV-5	*Herpesviridae*	[172]
Dimitroulia *et al.*, 2013	PCR	Gastric tissue	IBD	HHV-4	*Herpesviridae*	[173]
Dimberg *et al.*, 2013	PCR	Colon/rectum tissue	CRC	HHV-5	*Herpesviridae*	[174]
do Carmo *et al.*, 2014	PCR	Stools	IBD	HHV-5	*Herpesviridae*	[175]
El-Matary *et al.*,	PCR	Colon/rectum	IBD	HHV-5	*Herpesviridae*	[176]

2018		tissue				
Enam *et al.*, 2002	PCR/ISH	Colon/rectum tissue	CRC	JCV	*Polyomaviridae*	[177]
Esmailzadeh *et al.*, 2020	PCR	Colon/rectum tissue	CRC	JCV	*Polyomaviridae*	[178]
Eyre-Brook *et al.*, 1986	ISH	Colon/rectum tissue	IBD	HHV-5	*Herpesviridae*	[179]
Fahal *et al.*, 1995	Serology	Serum	GC	HIV, HBV	*Retroviridae, Hepadnaviridae*	[180]
Farmer *et al.*, 1973	Serology	Serum	IBD	Mumps, ADV, Reovirus,HHV-5, HHV-1, HHV-3	Epithelial, respiratory, intestinal, *Herpesviridae*	[181]
Fernandes *et al.*, 2019	Virome	Stool	IBD	*Caudovirales*	Phages	[50]
Fiorina *et al.*, 2014	PCR	Colon/rectum tissue	CRC	HHV-4	*Herpesviridae*	[182]
Flores *et al.*, 2014	PCR	Serum	IBD	BKV	*Polyomaviridae*	[183]
Gauss *et al.*, 2015	Record	—	IBD	HHV-5	*Herpesviridae*	[184]
Gazzaz *et al.*, 2016	PCR	Colon/rectum tissue	CRC	HPV	*Papillomaviridae*	[185]
Genitsch *et al.*, 2015	ISH	Gastric tissue	GC	HHV-4	*Herpesviridae*	[186]
Giuliani *et al.*, 2008	PCR	Colon/rectum tissue	CRC	SV40, JCV, BKV	*Polyomaviridae*	[187]
Goel *et al.*, 2006	PCR	Colon/rectum tissue	CRC	JCV	*Polyomaviridae*	[188]
Gong *et al.*, 2019	Record	—	IBD	HHV-4	*Herpesviridae*	[189]
Gonzalez *et al.*, 2015	Serology	Serum	CRC	HCV	*Flaviviridae*	[190]
Gordon *et al.*, 2016	Record	—	IBD	HHV-4	*Herpesviridae*	[191]
Gornick *et al.*, 2010	PCR	Colon/rectum tissue	CRC	HPV	*Papillomaviridae*	[192]
Green *et al.*, 1981	ISH	Colon/rectum tissue	CRC	HPV	*Papillomaviridae*	[193]
Greenberg *et al.*, 1978	Serology	Serum	IBD	Rotavirus	Intestinal	[194]
Grinstein *et al.*, 2002	ISH	Colon/rectum tissue	CRC	HHV-4	*Herpesviridae*	[195]
Gupta *et al.*, 2020	PCR	Colon/rectum tissue	CRC	HPV	*Papillomaviridae*	[196]
Haga *et al.*, 1996	PCR	Gastric tissue	IBD	Measles, Mumps, Rubella	Epithelial	[197]
Haghi-Navand *et al.*, 2019	PCR	Colon/rectum tissue	CRC	JCV	*Polyomaviridae*	[198]
Hamada *et al.*, 2014	Record	—	CRC	HIV	*Retroviridae*	[199]
Hampras *et al.*, 2014	Serology	Serum	CRC	JCV	*Polyomaviridae*	[200]
Hannigan *et al.*, 2018	Virome	Stool	CRC	*Caudovirales*	Phages	[52]
Harkins *et al.*, 2002	ISH	Colon/rectum tissue	CRC	HHV-5	*Herpesviridae*	[201]
Harsh *et al.*, 2017	Record	—	IBD	HBV, HCV, HIV	*Hepadnaviridae, Flaviviridae, Retroviridae*	[202]

Hart *et al.*, 1982	ISH	Colon/rectum tissue	CRC	HHV-5	*Herpesviridae*	[203]
Hayashi *et al.*, 1996	ISH	Gastric tissue	GC	HHV-4	*Herpesviridae*	[204]
He *et al.*, 2015	Serology	Serum	IBD	HBV	*Hepadnaviridae*	[205]
Hernandez-Losa *et al.*, 2003	PCR	Colon/rectum tissue	CRC	JCV	*Polyomaviridae*	[206]
Herrera-Goepfert *et al.*, 2005	ISH	Gastric tissue	GC	HHV-4	*Herpesviridae*	[207]
Hirata *et al.*, 2007	Record	—	CRC	HTLV-I	*Retroviridae*	[208]
Hori *et al.*, 2005	PCR	Colon/rectum tissue	CRC	JCV	*Polyomaviridae*	[209]
Hradsky *et al.*, 2015	PCR	Serum	IBD	HHV-4	*Herpesviridae*	[210]
Hsieh *et al.*, 1998	PCR	Gastric tissue	GC	HHV-4	*Herpesviridae*	[211]
Huang *et al.*, 1978	ISH	Colon/rectum tissue	CRC	HHV-5	*Herpesviridae*	[212]
Iizuka *et al.*, 2001	Serology	Serum	IBD	Mumps	Epithelial	[213]
Ito *et al.*, 1992	Serology	Serum	CRC	IMV	Epithelial	[214]
Jarzynski *et al.*, 2017	PCR	Colon/rectum tissue	CRC	HPV, BKV	*Papillomaviridae, Polyomaviridae*	[215]
Jung *et al.*, 2008	PCR	Colon/rectum tissue	CRC	JCV	*Polyomaviridae*	[216]
Jung *et al.*, 2019	Serology	Serum	CRC	HBV, HCV	*Hepadnaviridae, Flaviviridae*	[217]
Kambham *et al.*, 2004	ISH	Colon/rectum tissue	IBD	HHV-5	*Herpesviridae*	[218]
Kamiza *et al.*, 2016	Record	—	CRC	HBV, HCV	*Hepadnaviridae, Flaviviridae*	[219]
Kane *et al.*, 1971	Serology	Serum	IBD	HHV-4	*Herpesviridae*	[220]
Karbalaie-Niya *et al.*, 2018	PCR	Colon/rectum tissue	CRC	MCPV	*Polyomaviridae*	[221]
Karim *et al.*, 2003	ISH	Gastric tissue	GC	HHV-4	*Herpesviridae*	[222]
Karpinski *et al.*, 2011	PCR	Colon/rectum tissue	CRC	HHV-4, JCV, KSHV, ADV	*Herpesviridae, Polyomaviridae,* respiratory	[223]
Kattoor *et al.*, 2002	ISH	Gastric tissue	GC	HHV-4	*Herpesviridae*	[224]
Kawashima *et al.*, 2000	PCR	Serum	IBD	Measles	Epithelial	[225]
Kayamba *et al.*, 2016	Serology	Serum	GC	HHV-4	*Herpesviridae*	[226]
Keller *et al.*, 2014	Record	—	CRC	HIV	*Retroviridae*	[227]
Khabaz *et al.*, 2016b	PCR	Colon/rectum tissue	CRC	SV40	*Polyomaviridae*	[228]
Kiewe *et al.*, 2009	Serology	Serum	CRC	HHV-5	*Herpesviridae*	[229]
Kim *et al.*, 2010	ISH	Colon/rectum tissue	IBD	HHV-5	*Herpesviridae*	[230]
Kim *et al.*, 2014	Serology	Serum	IBD	HBV	*Hepadnaviridae*	[231]
Kirgan *et al.*, 1990	ISH	Colon/rectum tissue	CRC	HPV	*Papillomaviridae*	[99]
Kishore *et al.*, 2004	Serology	Serum	IBD	HHV-5	*Herpesviridae*	[232]
Knoell *et al.*, 2005	Serology	Serum	IBD	HBV, HCV	*Hepadnaviridae,*	[233]

					Flaviviridae	
Knosel *et al.*, 2004	PCR	Colon/rectum tissue	CRC	HHV-5	*Herpesviridae*	[234]
Knosel *et al.*, 2009	PCR	Colon/rectum tissue	IBD	HHV-4, HHV-5, HHV-1, HHV-6	*Herpesviridae*	[235]
Kocoglu *et al.*, 2016	Serology	Serum	CRC	HBV, HCV	*Hepadnaviridae, Flaviviridae*	[236]
Kojima *et al.*, 2006	Record	—	IBD	HHV-4	*Herpesviridae*	[237]
Kolho *et al.*, 2012	PCR	Stool	IBD	Norovirus	Intestinal	[238]
Kong *et al.*, 2007	PCR	Colon/rectum tissue	CRC	HPV	*Papillomaviridae*	[239]
Ksiaa *et al.*, 2010	PCR	Gastric tissue	GC	JCV	*Polyomaviridae*	[240]
Ksiaa *et al.*, 2015	PCR	Colon/rectum tissue	CRC	JCV	*Polyomaviridae*	[241]
Laghi *et al.*, 1999	PCR	Colon/rectum tissue	CRC	JCV	*Polyomaviridae*	[242]
Lavy *et al.*, 2001	Serology	Serum	IBD	Measles	Epithelial	[243]
Lee *et al.*, 2001	PCR	Colon/rectum tissue	CRC	HPV	*Papillomaviridae*	[244]
Leveque *et al.*, 2010	PCR	Colon/rectum tissue	IBD	HHV-5	*Herpesviridae*	[245]
Li *et al.*, 2007	ISH	Colon/rectum tissue	CRC	Parvo B19	Epithelial	[246]
Li *et al.*, 2019	PCR	Gastric tissue	IBD	HHV-4	*Herpesviridae*	[75]
Lin *et al.*, 2008	PCR	Colon/rectum tissue	CRC	JCV	*Polyomaviridae*	[247]
Liu *et al.*, 2011	PCR	Colon/rectum tissue	CRC	HPV	*Papillomaviridae*	[248]
Lu *et al.*, 2018	Serology	Serum	CRC	HBV	*Hepadnaviridae*	[249]
Lundstig *et al.*, 2007	Serology	Serum	CRC	JCV, BKV	*Polyomaviridae*	[250]
Lv *et al.*, 2020	Serology	Gastric tissue	IBD	HHV-5	*Herpesviridae*	[251]
MacKey *et al.*, 1979	ISH	Colon/rectum tissue	CRC	ADV	Respiratory	[252]
Maconi *et al.*, 2005	ISH	Colon/rectum tissue	IBD	HHV-5	*Herpesviridae*	[253]
Malekpour *et al.*, 2018	PCR	Colon/rectum tissue	CRC	HPV	*Papillomaviridae*	[254]
Mariguela *et al.*, 2008	PCR	Colon/rectum tissue	CRC/IBD	HHV-4, HHV-5	*Herpesviridae*	[255]
Mehrabani-Khasraghi *et al.*, 2016	PCR	Colon/rectum tissue	CRC	HHV-4, HHV-5, HHV-1	*Herpesviridae*	[256]
Militello *et al.*, 2009	PCR	Colon/rectum tissue	CRC	HHV-4, HHV-5, HPV	Multiple	[257]
Montgomery *et al.*, 1999	Record	—	IBD	Measles, Mumps, HHV-3	Epithelial	[258]
Morewaya *et al.*, 2004	ISH	Gastric tissue	GC	HHV-4	*Herpesviridae*	[259]
Mou *et al.*, 2012	PCR	Colon/rectum tissue	CRC	JCV	*Polyomaviridae*	[260]
Nakatsu *et al.*, 2018	Virome	Stool	CRC	Inovirus, Tunalikevirus,	Epithelial	[51]

				Bunyavirus		
Newcomb *et al.*, 2004	PCR	Colon/rectum tissue	CRC	JCV	*Polyomaviridae*	[261]
Niv *et al.*, 2010	PCR	Colon/rectum tissue	CRC	JCV	*Polyomaviridae*	[262]
Norman *et al.*, 2015	Virome	Stool	IBD	*Caudovirales*	Phages	[38]
Nosho *et al.*, 2009	PCR	Colon/rectum tissue	CRC	JCV	*Polyomaviridae*	[263]
Nosrati *et al.*, 2015	PCR	Colon/rectum tissue	CRC	HPV	*Papillomaviridae*	[264]
Oda *et al.*, 2003	ISH	Gastric tissue	GC	HHV-4	*Herpesviridae*	[265]
Perez *et al.*, 2005	PCR	Colon/rectum tissue	CRC	HPV	*Papillomaviridae*	[266]
Perez-Brocal *et al.*, 2013	Virome	Stool	IBD	*Caudovirales*	Phages	[48]
Perez-Brocal *et al.*, 2015	Virome	Stool	IBD	*Caudovirales*	Phages	[49]
Pironi *et al.*, 2009	PCR	Colon/rectum tissue	IBD	Parvo B19	Epithelial	[267]
Roblin *et al.*, 2011	ISH	Gastric tissue	IBD	HHV-5	*Herpesviridae*	[268]
Roblin *et al.*, 2012	Serology	Serum	IBD	HHV-5	*Herpesviridae*	[269]
Roche *et al.*, 1981	ISH	Colon/rectum tissue	CRC	HHV-5	*Herpesviridae*	[270]
Rollison *et al.*, 2009	PCR	Colon/rectum tissue	CRC	JCV	*Polyomaviridae*	[271]
Ruger *et al.*, 1985	ISH	Colon/rectum tissue	CRC	HHV-5	*Herpesviridae*	[272]
Ryan *et al.*, 2012	PCR	Gastric tissue	IBD	HHV-4	*Herpesviridae*	[73]
Salepci *et al.*, 2009	PCR	Colon/rectum tissue	CRC	HPV	*Papillomaviridae*	[273]
Samaka *et al.*, 2013	ISH	Colon/rectum tissue	CRC	JCV	*Polyomaviridae*	[274]
Sarvari *et al.*, 2018	PCR	Colon/rectum tissue	CRC	HHV-4, JCV	*Herpesviridae, Polyomaviridae*	[275]
Schildgen *et al.*, 2013	PCR	Colon/rectum tissue	CRC	Bocavirus	Respiratory	[276]
Shah *et al.*, 1992	PCR	Colon/rectum tissue	CRC	HPV	*Papillomaviridae*	[277]
Shibata *et al.*, 1993	PCR	Gastric tissue	GC	HHV-4	*Herpesviridae*	[278]
Sinagra *et al.*, 2014	PCR	Colon/rectum tissue	CRC	JCV	*Polyomaviridae*	[279]
Su *et al.*, 2020	Record	—	CRC	HBV	*Hepadnaviridae*	[280]
Sura *et al.*, 2010	PCR	Gastric tissue	IBD	HHV6	*Herpesviridae*	[281]
Tafvizi *et al.*, 2014	PCR	Colon/rectum tissue	CRC	HHV-5	*Herpesviridae*	[282]
Tafvizi *et al.*, 2015	PCR	Colon/rectum tissue	CRC	HHV-4	*Herpesviridae*	[283]
Taherian *et al.*, 2014	PCR	Colon/rectum tissue	CRC	HPV	*Papillomaviridae*	[284]
Theodoropoulos *et al.*, 2005	PCR	Colon/rectum tissue	CRC	JCV	*Polyomaviridae*	[285]
Tolentino *et al.*,	Serology	Serum	IBD	HBV	*Hepadnaviridae*	[286]

2008						
Toumi *et al.*, 2017	PCR	Colon/rectum tissue	CRC	JCV	*Polyomaviridae*	[287]
Trimeche *et al.*, 2009	PCR	Gastric tissue	GC	HHV-4	*Herpesviridae*	[288]
Truong *et al.*, 2009	ISH	Gastric tissue	GC	HHV-4	*Herpesviridae*	[289]
Tsai *et al.*, 2015	Record	—	IBD	HHV-3	*Herpesviridae*	[290]
Ungaro *et al.*, 2019	Virome	Record	IBD	Multiple herpersviruses	*Herpesviridae*	[54]
Vanoli *et al.*, 2017	ISH	Gastric tissue	IBD	HHV-4	*Herpesviridae*	[291]
Vega *et al.*, 1999	ISH	Gastric tissue	IBD	HHV-5	*Herpesviridae*	[292]
Vilkin *et al.*, 2012	ISH	Colon/rectum tissue	CRC	JCV	*Polyomaviridae*	[293]
Wagner *et al.*, 2013	Virome	Gastric tissue	IBD	*Caudovirales*	Phages	[53]
Wakefield *et al.*, 1992	PCR	Gastric tissue	IBD	HHV-4, HHV-5, HHV-6	*Herpesviridae*	[294]
Weinreb *et al.*, 2006	ISH	Colon/rectum tissue	CRC	JCV	*Polyomaviridae*	[295]
Wong *et al.*, 2003	ISH	Colon/rectum tissue	CRC	HHV-4	*Herpesviridae*	[296]
Yanai *et al.*, 1997	ISH	Gastric tissue	GC	HHV-4	*Herpesviridae*	[297]
Yanai *et al.*, 1999	ISH	Colon/rectum tissue	IBD	HHV-4	*Herpesviridae*	[298]
Yavuzer *et al.*, 2011	PCR	Colon/rectum tissue	CRC	HPV	*Papillomaviridae*	[299]
Yi *et al.*, 2013	PCR/Serology	Serum	IBD	HHV-5	*Herpesviridae*	[300]
Yuen *et al.*, 1994	ISH	Colon/rectum tissue	CRC	HHV-4	*Herpesviridae*	[301]
Yunos *et al.*, 2006	ISH	Gastric tissue	GC	HHV-4	*Herpesviridae*	[302]
Zagorowicz *et al.*, 2016	ISH	Colon/rectum tissue	IBD	HHV-5	*Herpesviridae*	[303]
Zapatka *et. al.*, 2020	Virome	Gastric and Colon/rectum tissues	GC	HHV-4<br>HBV	*Herpesviridae*<br>*Hepadnaviridae*	[55]

Table S2. NOS scores of the studies included in the present meta-analysis, including quantitative and qualitative assessment.

Author	Representativeness	Control group	Documentation	Outcome	Absence of co-infections	Score
Aarnio *et al.*, 2012	No	No	Yes	Yes	Yes	Moderate
Abdel-Moneim *et al.*, 2016	Yes	No	Yes	Yes	Yes	Moderate
Abdirad *et al.*, 2007	Yes	No	Yes	Yes	No	Moderate
Adams *et al.*, 2016	Yes	No	Yes	Yes	Yes	Moderate
Afzal *et al.*, 2000	No	No	Yes	Yes	Yes	Moderate
Afzal *et al.*, 1998	No	Yes	Yes	Yes	Yes	Moderate
Aghakhani *et al.*, 2014	Yes	No	Yes	Yes	Yes	Moderate
Ahmad *et al.*, 2019	Yes	No	Yes	Yes	Yes	Moderate
Akintola-Ogunremi *et al.*, 2005	Yes	Yes	No	Yes	Yes	Moderate
Alacam *et al.*, 2021	Yes	No	Yes	Yes	Yes	Moderate
Alain *et al.*, 2005	Yes	No	Yes	Yes	Yes	Moderate
Alipov *et al.*, 2005	Yes	No	Yes	Yes	Yes	Moderate
Audeau *et al.*, 2002	No	No	Yes	Yes	Yes	Moderate
Axelrad *et al.*, 2018	Yes	Yes	Yes	Yes	Yes	High
Axelrad *et al.*, 2019	Yes	Yes	Yes	Yes	Yes	High
Balzola *et al.*, 1998	Yes	Yes	Yes	Yes	Yes	High
Baran *et al.*, 2018	No	No	Yes	Yes	Yes	Moderate
Bellaguarda *et al.*, 2015	Yes	No	Yes	Yes	Yes	Moderate
Bender *et al.*, 2009	Yes	No	Yes	Yes	Yes	Moderate
Bernabe-Dones *et al.*, 2016	Yes	Yes	Yes	Yes	Yes	High
Bernstein *et al.*, 2007	Yes	Yes	Yes	Yes	Yes	High
Bertalot *et al.*, 2001	No	No	Yes	Yes	No	Low
Biancone *et al.*, 2001	Yes	Yes	Yes	Yes	Yes	High
Bodaghi *et al.*, 2005	Yes	Yes	Yes	Yes	Yes	High
Boltin *et al.*, 2010	No	Yes	Yes	Yes	Yes	Moderate
Brichacek *et al.*, 1980	No	Yes	Yes	Yes	Yes	Moderate
Burnett-Hartman *et al.*, 2011	Yes	No	Yes	Yes	Yes	Moderate
Burnett-Hartman *et al.*, 2012	Yes	Yes	No	Yes	Yes	Moderate
Burnett-Hartman *et al.*, 2013	Yes	Yes	Yes	Yes	Yes	High
Butt *et al.*, 2016	Yes	Yes	Yes	Yes	Yes	High
Campello *et al.*, 2010	Yes	Yes	Yes	Yes	Yes	High
Campello *et al.*, 2011	Yes	Yes	Yes	Yes	Yes	High
Cardenas-Mondragon *et al.*, 2015b	Yes	Yes	Yes	Yes	Yes	High
Carrascal *et al.*, 2003	Yes	No	Yes	Yes	Yes	Moderate
Casini *et al.*, 2005	No	Yes	No	Yes	Yes	Moderate
Chan *et al.*, 2016	Yes	No	Yes	Yes	Yes	Moderate
Chen *et al.*, 2016	Yes	No	No	Yes	Yes	Moderate
Chen *et al.*, 2017	Yes	No	Yes	Yes	No	Moderate
Chevaux *et al.*, 2010	Yes	No	Yes	Yes	Yes	Moderate
Cho *et al.*, 2001	Yes	No	Yes	Yes	Yes	Moderate
Coelho *et al.*, 2013	Yes	Yes	Yes	Yes	Yes	High
Cohen *et al.*, 2018b	Yes	No	Yes	Yes	Yes	Moderate
Dalla Libera *et al.*, 2020	Yes	Yes	Yes	Yes	No	Moderate
Damin *et al.*, 2007	Yes	No	Yes	Yes	Yes	Moderate
De Francisco *et al.*, 2018	Yes	No	Yes	Yes	Yes	Moderate
de Lima *et al.*, 2012b	Yes	No	Yes	Yes	No	Moderate
de Saussure *et al.*, 2004	Yes	No	Yes	Yes	Yes	Moderate
de Souza *et al.*, 2018	Yes	No	Yes	Yes	Yes	Moderate
de Villiers *et al.*, 2002	No	No	Yes	Yes	Yes	Moderate
de Villiers *et al.*, 2007	Yes	Yes	Yes	Yes	Yes	High

Del Moral-Hernandez *et al.*, 2019	No	No	Yes	Yes	Yes	Moderate
Deschoolmeester *et al.*, 2010	Yes	No	Yes	Yes	Yes	Moderate
Dimitroulia *et al.*, 2006	Yes	Yes	No	Yes	Yes	Moderate
Dimitroulia *et al.*, 2013	Yes	Yes	Yes	Yes	No	Moderate
Dinberg *et al.*, 2013	Yes	No	Yes	Yes	Yes	Moderate
do Carmo *et al.*, 2014	Yes	No	Yes	Yes	Yes	Moderate
El-Matary *et al.*, 2018	Yes	No	Yes	Yes	Yes	Moderate
Enam *et al.*, 2002	No	No	No	Yes	Yes	Low
Esmailzadeh *et al.*, 2020	Yes	Yes	Yes	Yes	Yes	High
Eyre-Brook *et al.*, 1986	No	No	Yes	Yes	Yes	Moderate
Fahal *et al.*, 1995	Yes	Yes	Yes	Yes	No	Moderate
Farmers *et al.*, 1973	Yes	Yes	Yes	Yes	Yes	High
Fernandes *et al.*, 2019	No	Yes	Yes	Yes	Yes	Moderate
Fiorina *et al.*, 2014	Yes	Yes	Yes	Yes	Yes	High
Flores *et al.*, 2014	Yes	Yes	Yes	Yes	Yes	High
Gauss *et al.*, 2015	Yes	No	Yes	Yes	Yes	Moderate
Gazzaz *et al.*, 2016	Yes	No	Yes	Yes	Yes	Moderate
Genitsch *et al.*, 2015	Yes	No	Yes	Yes	Yes	Moderate
Giuliani *et al.*, 2008	Yes	No	Yes	Yes	Yes	Moderate
Goel *et al.*, 2006	Yes	No	No	Yes	Yes	Moderate
Gong *et al.*, 2019	Yes	No	Yes	Yes	Yes	Moderate
Gonzalez *et al.*, 2015	Yes	No	Yes	Yes	Yes	Moderate
Gordon *et al.*, 2016	Yes	No	Yes	Yes	Yes	Moderate
Gornick *et al.*, 2010	Yes	Yes	Yes	Yes	Yes	High
Green *et al.*, 1981	Yes	Yes	Yes	Yes	Yes	High
Greenberg *et al.*, 1978	Yes	Yes	Yes	Yes	Yes	High
Grinstein *et al.*, 2002	No	No	Yes	Yes	Yes	Moderate
Gupta *et al.*, 2020	Yes	No	Yes	Yes	Yes	Moderate
Haga *et al.*, 1996	No	Yes	Yes	Yes	Yes	Moderate
Haghi-Navand *et al.*, 2019	Yes	Yes	Yes	Yes	Yes	High
Hamada *et al.*, 2014	Yes	Yes	Yes	Yes	No	Moderate
Hampras *et al.*, 2014	Yes	Yes	No	No	Yes	Moderate
Hannigan *et al.*, 2018	Yes	Yes	No	Yes	Yes	Moderate
Harkins *et al.*, 2002	No	Yes	Yes	Yes	Yes	Moderate
Harsh *et al.*, 2017	Yes	Yes	Yes	Yes	No	Moderate
Hart *et al.*, 1982	No	No	Yes	Yes	Yes	Moderate
Hayashi *et al.*, 1996	Yes	No	Yes	Yes	Yes	Moderate
He *et al.*, 2015	Yes	Yes	Yes	Yes	Yes	High
Hernandez-Losa *et al.*, 2003	No	Yes	Yes	Yes	Yes	Moderate
Herrera-Goepfert *et al.*, 2005	Yes	No	Yes	Yes	Yes	Moderate
Hirata *et al.*, 2007	Yes	Yes	Yes	Yes	Yes	High
Hori *et al.*, 2005	Yes	Yes	No	Yes	Yes	Moderate
Hradsky *et al.*, 2015	Yes	No	Yes	Yes	Yes	Moderate
Hsieh *et al.*, 1998	Yes	No	Yes	Yes	Yes	Moderate
Huang *et al.*, 1978	No	Yes	Yes	Yes	Yes	Moderate
Iizuka *et al.*, 2001	No	Yes	Yes	Yes	Yes	Moderate
Ito *et al.*, 1992	Yes	Yes	Yes	Yes	Yes	High
Jarzynski *et al.*, 2017	Yes	No	Yes	Yes	Yes	Moderate
Jung *et al.*, 2008	Yes	No	Yes	Yes	Yes	Moderate
Jung *et al.*, 2019	Yes	No	Yes	Yes	Yes	Moderate
Kambham *et al.*, 2004	Yes	Yes	Yes	Yes	Yes	High
Kamiza *et al.*, 2016	Yes	Yes	Yes	Yes	Yes	High
Kane *et al.*, 1971	Yes	Yes	Yes	Yes	Yes	High
Karbalaie-Niya *et al.*, 2018	Yes	Yes	Yes	Yes	Yes	High
Karim *et al.*, 2003	Yes	No	Yes	Yes	Yes	Moderate
Karpinski *et al.*, 2011	Yes	No	Yes	Yes	Yes	Moderate

Kattoor *et al.*, 2002	Yes	No	Yes	Yes	Yes	Moderate
Kawashima *et al.*, 2000	No	Yes	Yes	Yes	Yes	Moderate
Kayamba *et al.*, 2016	Yes	Yes	Yes	Yes	Yes	High
Keller *et al.*, 2014	Yes	No	No	Yes	Yes	Moderate
Khabaz *et al.*, 2016	Yes	Yes	Yes	Yes	Yes	High
Kiewe *et al.*, 2009	Yes	Yes	Yes	Yes	Yes	High
Kim *et al.*, 2010	Yes	Yes	Yes	Yes	Yes	High
Kim *et al.*, 2014	Yes	Yes	Yes	Yes	Yes	High
Kirgan *et al.*, 1990	Yes	Yes	Yes	Yes	Yes	High
Kishore *et al.*, 2004	Yes	No	Yes	Yes	Yes	Moderate
Knoell *et al.*, 2005	Yes	Yes	Yes	Yes	Yes	High
Knosel *et al.*, 2004	Yes	No	Yes	Yes	Yes	Moderate
Knosel *et al.*, 2009	Yes	Yes	Yes	Yes	Yes	High
Kocoglu *et al.*, 2016	Yes	Yes	Yes	Yes	Yes	High
Kojima *et al.*, 2006	No	No	Yes	Yes	Yes	Moderate
Kolho *et al.*, 2012	Yes	Yes	No	Yes	Yes	Moderate
Kong *et al.*, 2007	No	No	Yes	Yes	Yes	Moderate
Ksiaa *et al.*, 2010	Yes	Yes	Yes	Yes	Yes	High
Ksiaa *et al.*, 2015	Yes	No	Yes	Yes	Yes	Moderate
Laghi *et al.*, 1999	No	No	No	Yes	Yes	Low
Lavy *et al.*, 2001	Yes	Yes	Yes	Yes	Yes	High
Lee *et al.*, 2001	No	Yes	Yes	Yes	Yes	Moderate
Leveque *et al.*, 2010	Yes	No	Yes	Yes	Yes	Moderate
Li *et al.*, 2007	Yes	Yes	Yes	Yes	Yes	High
Li *et al.*, 2019	Yes	Yes	Yes	Yes	Yes	High
Lin *et al.*, 2008	No	No	Yes	Yes	Yes	Moderate
Liu *et al.*, 2011	Yes	Yes	Yes	Yes	Yes	High
Lu *et al.*, 2018	Yes	No	Yes	Yes	Yes	Moderate
Lundstig *et al.*, 2007	No	No	Yes	Yes	Yes	Moderate
Lv *et al.*, 2020	Yes	Yes	Yes	Yes	Yes	High
MacKey *et al.*, 1979	No	No	Yes	Yes	Yes	Moderate
Maconi *et al.*, 2005	Yes	Yes	Yes	Yes	Yes	High
Malekpour *et al.*, 2018	Yes	No	Yes	Yes	Yes	Moderate
Mariguela *et al.*, 2008	No	No	Yes	Yes	Yes	Moderate
Mehrabani-Khasraghi *et al.*, 2016	No	Yes	Yes	Yes	Yes	Moderate
Militello *et al.*, 2009	Yes	Yes	Yes	Yes	Yes	High
Montgomery *et al.*, 1999	Yes	Yes	Yes	Yes	Yes	High
Morewaya *et al.*, 2004	Yes	No	Yes	Yes	Yes	Moderate
Mou *et al.*, 2012	Yes	Yes	No	No	Yes	Moderate
Nakatsu *et al.*, 2018	Yes	Yes	No	Yes	Yes	Moderate
Newcomb *et al.*, 2004	Yes	Yes	Yes	Yes	Yes	High
Niv *et al.*, 2010	No	No	No	No	Yes	Low
Norman *et al.*, 2015	Yes	Yes	Yes	Yes	Yes	High
Nosho *et al.*, 2009	Yes	No	Yes	Yes	Yes	Moderate
Nosrati *et al.*, 2015	Yes	Yes	No	No	Yes	Moderate
Oda *et al.*, 2003	Yes	No	Yes	Yes	Yes	Moderate
Perez *et al.*, 2005	Yes	Yes	Yes	Yes	Yes	High
Perez-Brocal *et al.*, 2013	No	Yes	No	No	Yes	Low
Perez-Brocal *et al.*, 2015	No	Yes	Yes	Yes	Yes	Moderate
Pironi *et al.*, 2009	No	No	Yes	Yes	No	Low
Roblin *et al.*, 2011	No	No	Yes	Yes	Yes	Moderate
Roblin *et al.*, 2012	Yes	No	Yes	Yes	Yes	Moderate
Roche *et al.*, 1981	No	No	No	Yes	Yes	Low
Rollison *et al.*, 2009	Yes	Yes	Yes	Yes	Yes	High
Ruger *et al.*, 1985	No	No	Yes	Yes	Yes	Moderate
Ryan *et al.*, 2012	No	Yes	Yes	Yes	Yes	Moderate

Salepci *et al.*, 2009	Yes	Yes	Yes	Yes	Yes	High
Samaka *et al.*, 2013	Yes	Yes	No	Yes	Yes	Moderate
Sarvari *et al.*, 2018	Yes	Yes	Yes	Yes	Yes	High
Schildgen *et al.*, 2013	No	No	Yes	Yes	Yes	Moderate
Shah *et al.*, 1992	Yes	No	Yes	Yes	Yes	Moderate
Shibata *et al.*, 1993	Yes	No	Yes	Yes	Yes	Moderate
Sinagra *et al.*, 2014	No	Yes	Yes	Yes	Yes	Moderate
Su *et al.*, 2020	Yes	Yes	Yes	Yes	Yes	High
Sura *et al.*, 2010	No	Yes	Yes	Yes	Yes	Moderate
Tafvizi *et al.*, 2014	Yes	Yes	Yes	Yes	Yes	High
Tafvizi *et al.*, 2015	Yes	Yes	Yes	Yes	Yes	High
Taherian *et al.*, 2014	Yes	Yes	Yes	Yes	Yes	High
Theodoropoulos *et al.*, 2005	Yes	Yes	Yes	Yes	Yes	High
Tolentino *et al.*, 2008	Yes	No	Yes	Yes	Yes	Moderate
Toumi *et al.*, 2017	No	No	Yes	Yes	Yes	Moderate
Trimeche *et al.*, 2009	Yes	No	Yes	Yes	Yes	Moderate
Truong *et al.*, 2009	Yes	No	Yes	Yes	Yes	Moderate
Tsai *et al.*, 2015	No	Yes	No	No	Yes	Low
Ungaro *et al.*, 2019	No	No	Yes	Yes	Yes	Moderate
Vanoli *et al.*, 2017	No	No	Yes	Yes	Yes	Moderate
Vega *et al.*, 1999	No	No	Yes	Yes	Yes	Moderate
Vilkin *et al.*, 2012	Yes	Yes	No	No	Yes	Moderate
Wagner *et al.*, 2013	No	No	No	Yes	Yes	Low
Wakefield *et al.*, 1992	Yes	Yes	Yes	Yes	Yes	High
Weinreb *et al.*, 2006	Yes	No	Yes	Yes	Yes	Moderate
Wong *et al.*, 2003	No	No	Yes	Yes	Yes	Moderate
Yanai *et al.*, 1997	No	No	Yes	Yes	Yes	Moderate
Yanai *et al.*, 1999	No	Yes	Yes	Yes	Yes	Moderate
Yavuzer *et al.*, 2011	Yes	Yes	No	Yes	Yes	Moderate
Yi *et al.*, 2013	Yes	Yes	Yes	Yes	Yes	High
Yuen *et al.*, 1994	No	No	Yes	Yes	Yes	Moderate
Yunos *et al.*, 2006	No	No	Yes	Yes	Yes	Moderate
Zagorowicz *et al.*, 2016	Yes	No	Yes	Yes	Yes	Moderate
Zapatka *et al.*, 2020	No	No	Yes	Yes	Yes	Moderate
